# QSAR Modeling on Benzo[*c*]phenanthridine Analogues as Topoisomerase I Inhibitors and Anti-cancer Agents

**DOI:** 10.3390/molecules17055690

**Published:** 2012-05-11

**Authors:** Khac-Minh Thai, Quang-Huynh Bui, Thanh-Dao Tran, Thi-Ngoc-Phuong Huynh

**Affiliations:** Department of Medicinal Chemistry, School of Pharmacy, University of Medicine and Pharmacy at Ho Chi Minh City, 41 Dinh Tien Hoang St., Dist. 1, Ho Chi Minh City, Vietnam; E-Mails: quanghuynhct@gmail.com (Q.-H.B.); thanhdaot@yahoo.com (T.-D.T.); ngocphuonght@yahoo.com (T.-N.-P.H.)

**Keywords:** QSAR, topoisomerase, benzo[c]phenanthridine, cytotoxicity, model assessment, confidence level

## Abstract

Benzo[*c*]phenanthridine (BCP) derivatives were identified as topoisomerase I (TOP-I) targeting agents with pronounced antitumor activity. In this study, hologram-QSAR, 2D-QSAR and 3D-QSAR models were developed for BCPs on topoisomerase I inbibitory activity and cytotoxicity against seven tumor cell lines including RPMI8402, CPT-K5, P388, CPT45, KB3-1, KBV-1and KBH5.0. The hologram, 2D, and 3D-QSAR models were obtained with the square of correlation coefficient R^2^ = 0.58 − 0.77, the square of the crossvalidation coefficient q^2^ = 0.41 − 0.60 as well as the external set’s square of predictive correlation coefficient r^2^ = 0.51 − 0.80. Moreover, the assessment method based on reliability test with confidence level of 95% was used to validate the predictive power of QSAR models and to prevent over-fitting phenomenon of classical QSAR models. Our QSAR model could be applied to design new analogues of BCPs with higher antitumor and topoisomerase I inhibitory activity.

## 1. Introduction

**Figure 1:**
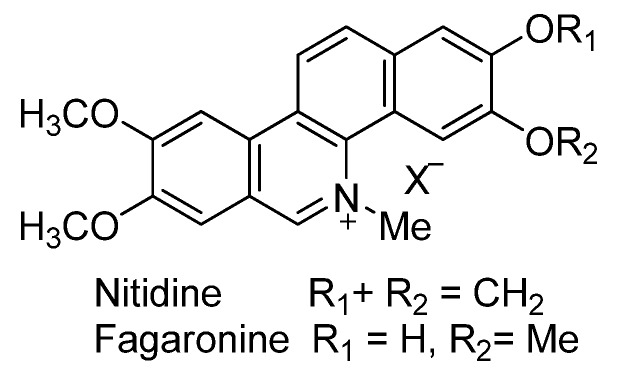
Structure of nitidine and fagaronine.

The topoisomerases (TOP) are enzymes involved in DNA replication, repair, transcription, recombination and segregation. The DNA topoisomerase I (TOP-I) is considered as one of the most effective targets for developing anti-cancer agents, not only due to its abnormally high intracellular levels, but also by the restriction of corrective mechanisms’ cleavage of stabled TOP-I-DNA [1,2]. Among the groups showing the resistance to TOP-I activity, the substances similar to benzo[*c*]phenanthridine synthesized by Lavoie and colleagues (Figure 1) have shown significant cytotoxicity [3,4,5,6,7,8,9,10,11]. Although over 130 compounds have been synthesized but the QSAR studies on this group are still rare and its application is limited [12,13].

In this study, a dataset of 137 benzo[*c*]phenanthridine (BCP) analogues with TOP-I inhibitory activity and antitumor activity against seven cell lines, including RPMI8402, CPT-K5, P388, CPT45, KB3-1, KBV-1 and KBH5.0, were chosen for hologram-QSAR (H-QSAR), 2D-QSAR as well as 3D-QSAR studies with CoMFA and CoMSIA analyses. By combining three QSAR methods, we expect that the theoretical results can decrease the error of the prediction and offer some useful information for designing and screening more potential antitumor compounds with less time and cost.

## 2. Result and Discussion

### 2.1. The Benzo[c]phenanthridins and Their Biological Activity Data

**Figure 2:**
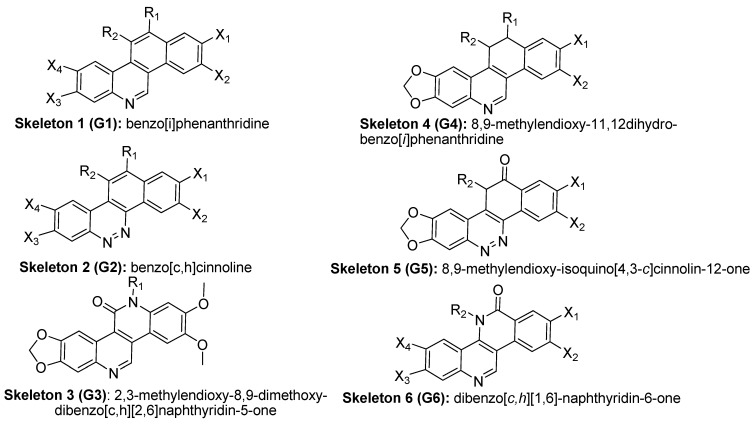
General structural skeletons of the BCPs dataset.

The compounds studied in this work were BCP derivatives having a similar core to the two alkaloids nitidine and fagaronine shown in Figure 1 [1,14]. The *in vitro* TOP-I inhibition data (REC, which is the relative effective concentration of TOP-I related to topotecan) and IC_50_ values (the concentration of compound causing 50% cell growth inhibition against tumor cell lines) on RPMI8402, CPT-K5, P388, CPT45, KB3-1, KBV-1, KBH5.0, U937 and U937rs of 137 chemical structures related to BCPs were collected from the literature [3,4,5,6,7,8,9,10,11]. However, not all bioactivity data of different cell lines is available for each compound. Compounds numbers and available bioactivity data are listed in Table 1. U937 and U937rs cell lines having a limited number of cytotoxicity data were not used for developing the QSAR model. REC and IC_50_ values were converted to negative logarithm of REC, IC_50_ (pREC, pIC50) for use in the QSAR studies. Chemically, the dataset can be divided into six groups of general skeletons presented in Figure 2 and the number of compounds of each group are shown in Table 1. The number of compounds in the training and external test sets are presented in Table 2. The detailed chemical structures and bioactivity of BCP dataset are presented in the Supporting Information.

### 2.2. Over-fitting Problem

**Figure 3:**
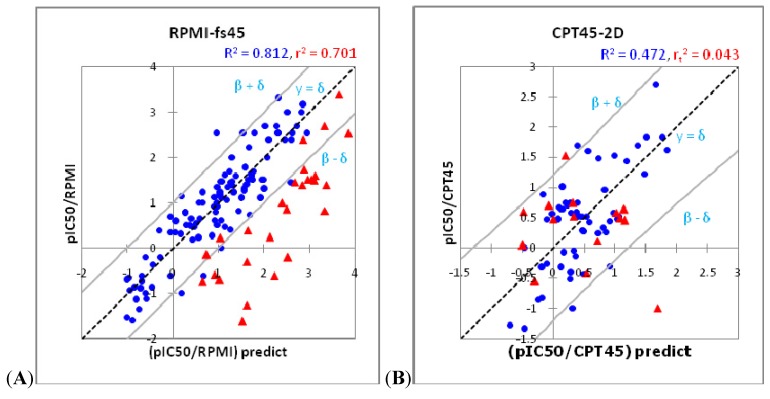
The relationship between observed and predicted data from QSAR model and its 95% confidence interval of (**A**) RPMI8402 cell line from 3D QSAR with steric analysis fields and (**B**) CPT45 cell line from 2D QSAR. Compound of training set are in blue circle and test set in red triangle.

A well-accepted QSAR model should be able to accurately predict activities of a new compound which is not included in the training set. Over-fitting or over-estimation occurs when the predictive ablility on external set is bad, some papers use r^2^ for this assessment [15,16]. However, using the square of correlation coefficient is not exact in all cases and cannot manifest the meaning of the model predictions. In this study, the results of 3D QSAR model of RPMI8402 cell line and 2D QSAR model of CPT45 cell line were given as examples. Accordingly, RPMI-fs45 model (R^2^ = 0.812 for training set; r^2^ = 0.701 for test set) was assessed as having a good result and the ability to predict accurately but in fact the predictive power of this model is bad (Figure 3A) with the external set of model RPMI-fs45 (red triangle) tending to go out of the two limit lines at a confidence level of 95% of training set (blue circle), whereas, the CPT45-2D model gave a reasonable result based on the 95% confidence level assessment method, which is shown in Figure 3B. Hence, the QSAR model with high value r^2^ of training and test sets does not necessarily correlate with a good predictive model.

### 2.3. Model Assessment Method

For QSAR validation, several parameters such as R^2^, q^2^, standard error of training and test sets, Y-scrambling analyses, and confidence interval estimators were used to judge the QSAR models [12,13,16,17,18,19,20]. Confidence level is the result of statistical estimation based on observations on a population. This estimated level is hard to reach 100%, therefore, the statisticians often use the estimate of 90%, 95%, 99% confidence intervals [15,18]. For classical QSAR study, 95% confidence interval is commonly used as the parameter in validation of QSAR models. In this study, the QSAR model evaluation method based on confidence level is presented as below.

At a confidence level of 95%, the limit is calculated so that 95% of training is in the area limited by the upper and lower bounds as shown in Figure 3. The two bounds are almost straight lines parallel to the baseline y = x. If the two bounds meet the horizontal axis at the points x1= −x2= δ (δ > 0), they can be assumed as two lines y = x − δ and y = x + δ. The d value represents the desired predictability of the model which depends on squared correlation coefficient R^2^ and standard error of the predicted results compared with experimental values of training set. If the model gave the predicted β, then 95% of β was in the range β ± δ.

The model predictions are confirmed as true to its ability when the external evaluation set with coordinates (x_i_ = pIC50 expected, y_i_ = pIC50 experimental) lies within the boundaries of the two lines, the type I error probability is 5% (if the external set is large enough, there will be 5% of compounds with the predicted value lies outside the confidence interval).

The assessment step in this study is done as follows:

- Determine the δ from the training set.

- Use a set of external set to assess the reliability of the value of δ. The reliability of the value of δ is evaluated by seeing how much the coordinates of the compounds in the external set properly distributed in the confidence limits. This number is not required to be larger than or equal to the value of reliability (95%) but the difference of those two numbers must not be too large. The signs were used in this study for assessing the value δ with possitive (+), negative (−) and unknown (+/−).

- If r^2^ is also greater than 0.5, the model can be proposed to predict beyond the range of values evaluated.

In addition, several new metrics $r_{m}^{2}$, $\bar{r_{m}^{2}}$ and $\Delta r_{m}^{2}$ proposed by Roy’s reasearch group was also calculated for both training and test set to validate our QSAR models [21,22,23]. These additional validation parameters were used to assess the predictive quality of QSAR models. For the good QSAR models, the values of $\bar{r_{m}^{2}}$ should have be more than 0.5 and $\Delta r_{m}^{2}$ values should preferably be lower than 0.2 for both of the training and test sets. The equations for calculation of $r_{m}^{2}$, $\bar{r_{m}^{2}}$ and $\Delta r_{m}^{2}$ metrics could be found at supporting information.

### 2.4. Hologram, 2D and 3D QSAR Modeling

In this study, eight 2D QSAR models, eight hologram QSAR models and thirteen 3D QSAR models for TOP-I inhibitory activity and anti-toxicity on RPMI8402, CPT-K5, P388, CPT45, KB3-1, KBV-1, KBH5.0 tumor cell line were developed and the results are presented in Table 3 (2D), Table 4 (Hologram), Table 5 (3D) and the assessments of corresponding models with a confidence level of 95% are also presented. Based up on 95% confidence interval, the δ value, assessment and range of prediction of all obtained models were calculated. There are several models with good R^2^ and q^2^ values of training and test sets but those models could not give the predictive power for external test set by applying the confidence intervals (Table 5).

The models of RPMI-8402, KB3-1 cell lines and on TOP-I inhibitory activity have correlated and results in all three methods’ building QSAR models are reasonable. The hologram, 2D and 3D- QSAR models performed on pREC (topoisomerase inhibitory activity) and pIC50 of RPMI8402, KB3-1 cell-lines showed not only significant statistical quality, but also predictive ability, with the square of correlation coefficient R^2^ = 0.584 − 0.768, the square of the crossvalidation coefficient q^2^ = 0.406 − 0.594 as well as the external set’s square of predictive correlation coefficient r^2^ = 0.514 − 0.795. For RPMI 8402 cell line and KB3-1 cell-lines, the largest range of prediction are [−1:3] from hologram model and [−0.5:2.2] from 2D QSAR, respectively, were obtained. The best range of prediction for anti-topoisomerase 1 is [−2.5:1] is achieved from 3D QSAR model. Based on the calculation of $r_{m}^{2}$, $\bar{r_{m}^{2}}$ and $\Delta r_{m}^{2}$ metrics, several good QSAR models are highlighted in bold numbers in Table 3, Table 4 and Table 5. Detailed of 29 QSAR models are available in supporting information.

**Figure 4:**
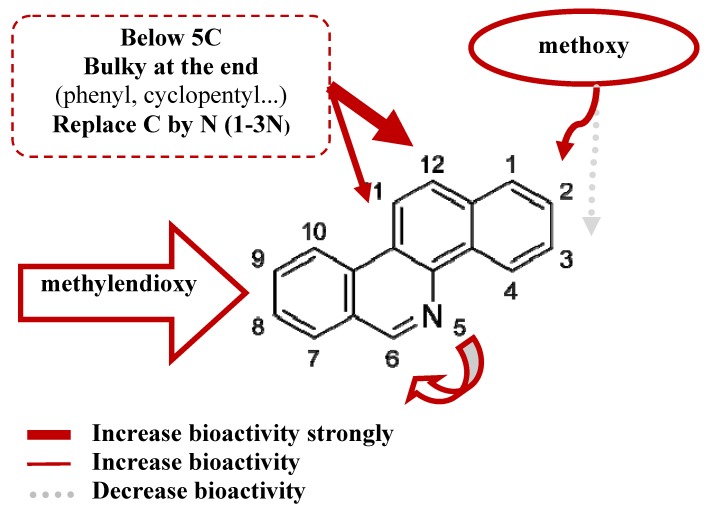
The summary for BCPs structures—antitumor activity relationship.

QSAR models on topoisomerase inhibitory activity and cytotoxicity of RPMI8402, KB3-1 cell-lines were used for further investigation on application set. The prediction on application set containing 1214 new virtual designed compounds offers a short list of 94 compounds with better predictive antitumor activity. Several selected compounds with predicted bioactive values are listed in supporting information. Analysis of the results from our QSAR models shows the general points of the relationship between chemical structures and antitumor activity of BCP derivatives summarized in Figure 4 and described as follows:

(1). The steric interaction plays an important role in determining the bioactivities of the BCP against many tumor cell lines, including cytotoxicity and TOP-I inhibitory ability. Substituents at 8,9-dimethoxy position on the skeletons are necessary for the biological effects. The results have shown that methoxy group at position 2 is essential for bioactivity while position 3 is not essential. The substituents at position 11, 12 affect the activity and should have a length of 4-5 carbons or lower, be straight up with the bulky end groups.
(2). Reducing the amount of nitrogen in the rings system and increasing the number of nitrogen atoms in the substituent can improve the bioactivity. Nitrogen in position 6 gave a better effect than position 5.
(3). The substituents at two positions 11 and 12 could have a positive effect on cytotoxicity and TOP-I inhibitory activity. The substituent at position 12 gives a stronger effect on bioactivity than position 11.

The previous studies indicated that topotecan, the synthetic derivative of camptothecin is the most potent anticancer drugs in clinical use [24]. Topotecan, ethoxidine, fagaronine and BCP related compounds indicated the selectivity on TOP-I than TOP-II. These novel compounds acted as DNA intercalators and have two mechanisms including *(i)* TOP-I poison activity like fagaronine; and *(ii)* TOP-I suppressor activity like ethoxidine [24,25]. Our preliminary results from *in silico* modeling indicated that BCP compounds may inhibit the TOP-I activity via suppression mechanism. From this QSAR study, the important role of natural functional groups related to biological activity is indicated in Figure 4. Hence, the combination of our QSAR models with other classification on TOP-I and cytotoxicity predictive models and molecular docking studies [12,25,26] could provide insight into the molecular basis of BCPs derivatives on antitumor and TOP-I inhibitory activity.

## 3. Materials and Methods

### 3.1. QSAR Study Process

**Figure 5:**
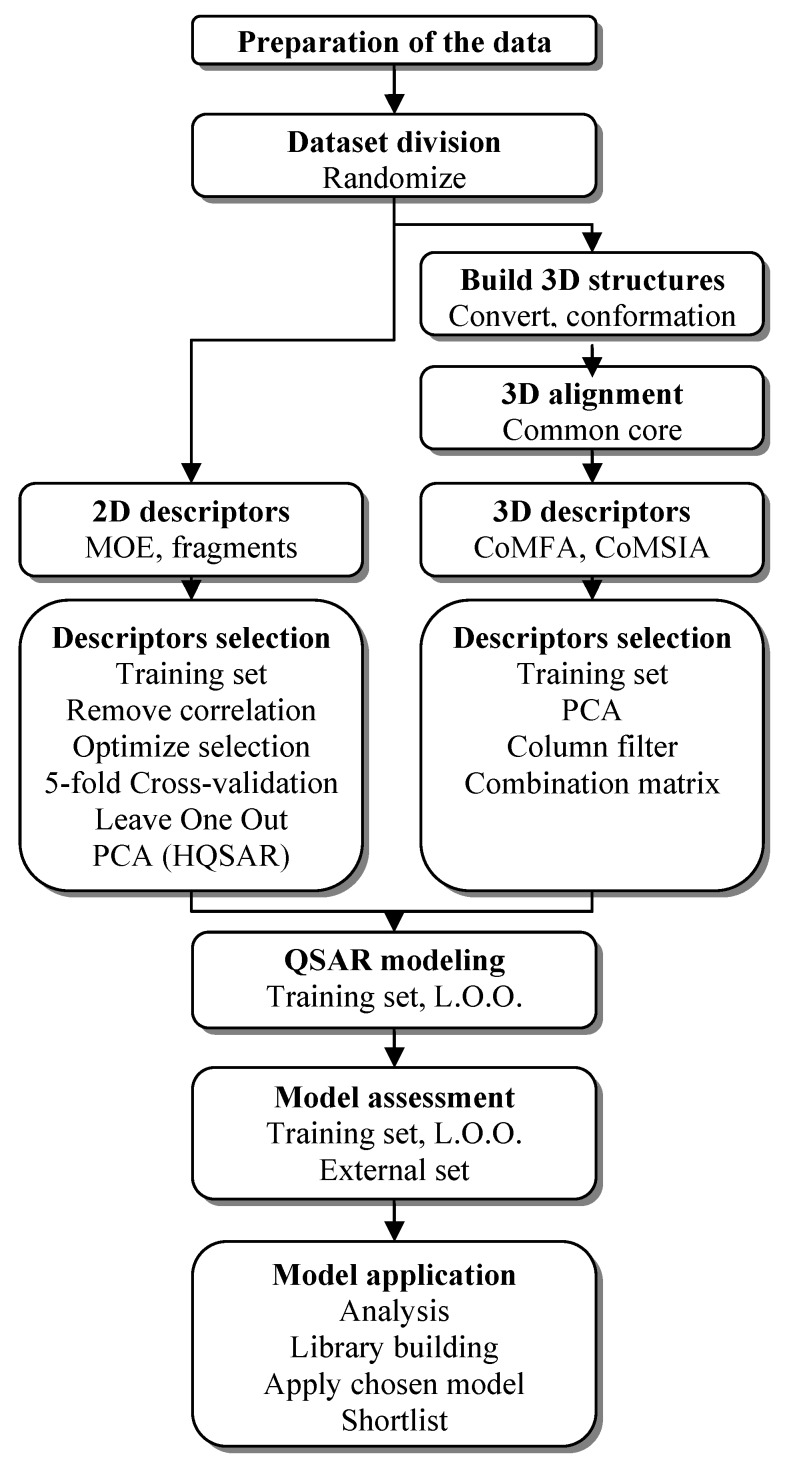
Process of combined QSAR studies.

The QSAR study process is summarized in Figure 5.

### 3.2. Preparation of the Data Sets

A total of 137 chemical structures of benzo[*c*]phenanthridine analogues were collected from the literature (Table 6). The structures of the compounds were first drawn in Molecular Operating Environment sofware (MOE), named and put into global dataset [27]. The published data is direct copied and converted from *.pdf into a table in accordance with the format *.csv. Data will be imported into the used programs with the command “import”, “read”, “merge” based on the name of each subtance in order to ensure the precision and convenience. Recheck the drawn structures by using SAR report in MOE and Ligand Prepare in SYBYL-X 1.1 [28].

Using the SAR report generated by MOE, the dataset was devided into six groups according to structural skeleton similarity. Then we calculated the weight descriptors and sorted the compounds in order of molecular weight. The training and test sets were generated by random division using the original variable descriptors along with cytotoxicity and TOP-I inhibitory activity values. The data set is split randomly for five times into 80% for training and 20% for the test set and the results were presented in Table 2.

### 3.3. Hologram-QSAR

#### 3.3.1. Calculated Fragment Descriptors

Using all suggested descriptors but in order to save computational time, the limitation of number’s atoms must be set from 4–7 and change step by step from 1 to 10 [28].

#### 3.3.2. Hologram-QSAR Process

The standardized structures in 2D-QSAR were used in building Hologram-QSAR by SYBYL software. The importable files must be *.sdf, which are results from MOE. Changing the parameters of atoms’ limitation and doing Partial least squared regression with default principal components.

### 3.4. 2D-QSAR

#### 3.4.1. Calculated 2D-descriptors

The structures of the compounds were first standardized by “Depict2D” command then calculated of 184 2D-descriptors in MOE software [27].

#### 3.4.2. 2D-QSAR Process

The data was transferred to Rapidminer software for multi linear 2D-QSAR process [29]. The process has eight main steps which are shown in Figure S1 in the Supporting Information:

1^st^ step: Transfer data from MOE.

2^nd^ step: Remove useless descriptors with more than 20% compounds having value = 0.

3^rd^ step: Remove the desriptors with intercorrelation greater than 0.8.

4^th^ step: Optimize selection with modified forward selection using multi linear regression algorithm (MLR). The modifications include limiting of descriptors, keeping more than 1 best subset of descriptors and validating by Leave One Out (L.O.O.) cross-validation.

5^th^ step: Build the model using MLR and use L.O.O. to validate the predictive ability 

6^th^ step: Give the parameters on the training set.

7^th^ step: Give the parameters on the external set.

8^th^ step: Give the predictive results on application.

Several subset of chemical descriptors having an effect on the performance of predictition of anticancer and TOP-I inhibitory activity were selected and showed in supporting informtion for each QSAR models. The detailed of these molecular descriptors are described in Table 7.

### 3.5. 3D-QSAR

#### 3.5.1. Calculated 3D-descriptors

The stable conformation of the 3D structure is very important to develop a reliable and repetitive 3D-QSAR models. The search for lowest energy 3D conformations were conducted in MOE with forcefield MMFF94 and RMS Gradient 0.0001 kcal.mol^−1^. The results were transferred to SYBYL and MMFF94 charges were assigned to all the molecules [28].

**Figure 6:**
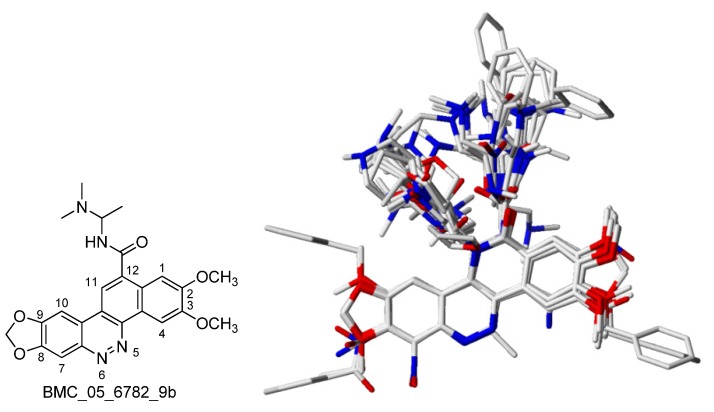
Structure of BMC_05_6782_9b and 3D alignment of 137 BCPs chemical structures.

Structural alignment is considered as one of the most sensitive parameters in CoMFA and CoMSIA analysis. The accuracy of the prediction and the reliability of the contour maps are directly dependent on the structural alignment rule. The compound BMC_05_6782_9b was used as a template for superimposition, the common fragment for each group was determined based on comparison with this compound’s core structure. The aligned compounds are shown in Figure 6.

Steric (fa) and electrostatic (fe) fields for CoMFA, steric (s), electrostatic (e), hydrogen bond donor (d), hydrogen bond acceptor (a) and hydrophobic descriptor (h) fields were calculated by using the default of SYBYL-X 1.1 with an sp^3^ carbon atom having *van der Waals* radius of 0.152nm, +1 charge, and 0.2 nm grid spacing. The energy cutoff values were set to 30 kcal.mol^−1^.

#### 3.5.2. 3D-QSAR Process

Each training set was conducted on 32 models with different 3D descriptors and a vary column filter values from 1 to 5. The PLS analysis was used to construct a linear correlation between the subset of descriptors and the bioactivities. To select the best model, the cross-validation L.O.O. was performed to reduce the square of crossvalidation coefficient (q^2^) and the optimum number of principal components. The q^2^ results were recorded into combination matrix table such as result on KB3-1 in Table 8. Detailed of q^2^ matrix of 3D models for diferrent cell lines could be found at supporting information.

## 4. Conclusions

In this study, the hologram, 2D- and 3D-QSAR analyses were used to build up the model for prediction of 137 BCP analogues based on their anti-topoisomerase-1 activity and cytotoxicity on seven tumor cell lines. The best model was obtained between pREC (topoisomerase inhibitory activity) and pIC50 of RPMI8402, KB3-1 cell-lines biological data and BCP analogues. In addition, the reliability test with 95% confidence interval was applied as a parameter for QSAR models validation on internal and external dataset and to prevent over-fitting problem of classical QSAR models. With its high accuracy and fast prediction on the BCPs, our QSAR model could be applied to design new analogues of BCPs with higher antitumor and topoisomerase I inhibitory activity.

## Figures and Tables

**Table 1:** Dataset and biological activities used in this study.

Bioactivity	Number of compounds	Number of compounds on each skeleton					
		G1	G2	G3	G4	G5	G6
RPMI	133	65	9	13	6	10	30
CPTk5	101	50	8	12	6	8	17
P388	82	53	1	4	5	9	10
CPT45	73	45	0	4	5	9	10
U937	39	23	9	0	6	0	1
U937rs	33	20	7	0	5	0	1
KB3-1	83	53	10	13	6	0	1
KBV-1	81	52	9	13	6	0	1
KBH	60	46	0	13	0	0	1
TOP-I	94	52	8	12	5	9	8

**Table 2:** Dataset division.

Bioactivity	RPMI 8402	CPT-K5	P388	CPT45	KB3-1	KBV-1	KBH5.0	TOP-I
Number of compounds	133	101	82	73	83	81	60	94
Training set	105	80	66	58	68	60	48	74
External test set	28	21	16	15	15	21	12	20

**Table 3:** Results of 2D-QSAR.

Model	RPMI	CPTk5	P388	CPT45	KB3-1	KBV	KBH	TOP-I
Number compounds in training set	105	80	66	58	68	60	48	74
Number compounds in external test set	28	21	16	15	15	21	12	20
R^2^ (Training set)	0.584	0.452	0.655	0.472	0.627	0.632	0.536	0.602
Standard Error (Training set)	0.543	0.400	0.271	0.338	0.414	0.248	0.218	0.355
q^2^ (L.O.O.)	0.511	0.302	0.417	0.230	0.537	0.474	0.394	0.475
Standard Error (L.O.O.)	0.641	0.522	0.489	0.527	0.520	0.364	0.29	0.477
r_t_^2^ (External set)	0.514	0.248	0.334	0.043	0.514	0.314	0.053	0.657
Standard Error (External set)	0.803	0.434	0.799	0.943	0.665	0.858	0.78	0.417
p-value of model	0.000	0.000	0.000	0.000	0.000	0.000	0.000	0.000
Number of 2D molecular descriptor	7	9	10	9	6	8	6	10
Greates p-value of used descriptors	0.039	0.018	0.014	0.000	0.026	0.002	0.026	0.008
**Model assessment**								
δ	1.45	1.30	1.10	1.20	1.25	1.00	1.00	1.25
Assessment	+	+	+	+	+	+	+	+
Range of prediction	−1.5 2.2	−1.2 1.4	0 2.3	−0.5 1.25	−0.5 2.2	−1.2 1.5	0.5 1.7	−2 0.5
$r_{m}^{2}$	**0.584**	0.452	0.655	0.472	**0.627**	0.632	0.536	**0.602**
$r_{m}^{/2}$	**0.399**	0.374	0.424	0.284	**0.433**	0.506	0.264	**0.451**
$\bar{r_{m}^{2}}$	**0.491**	0.413	0.540	0.378	**0.530**	0.569	0.400	**0.527**
$\Delta r_{m}^{2}$	**0.186**	0.078	0.231	0.188	**0.194**	0.126	0.272	**0.151**
$r_{m(test)}^{2}$	**0.514**	0.248	0.256	0.018	**0.591**	0.278	0.035	**0.588**
$r_{m(test)}^{/2}$	**0.324**	0.169	0.187	0.012	**0.549**	0.180	−0.007	**0.413**
$\bar{r_{m(test)}^{2}}$	**0.419**	0.208	0.222	0.015	**0.570**	0.229	0.014	**0.501**
$\Delta r_{m(test)}^{2}$	**0.190**	0.079	0.068	0.006	**0.042**	0.099	0.042	**0.175**

**Table 4:** Results of Hologram-QSAR.

Model	RPMI	CPTk5	P388	CPT45	KB3-1	KBV	KBH	TOP-I
Number compounds in training set	105	80	66	58	68	60	48	74
Number compounds in external test set	28	21	16	15	15	21	12	20
R^2^ (Training set)	0.765	0.573	0.439	0.483	0.622	0.501	0.624	0.616
Standard Error (Training set)	0.489	0.428	0.675	0.586	0.658	0.418	0.439	0.594
q^2^ (L.O.O.)	0.568	0.320	0.182	0.123	0.514	0.328	0.482	0.406
Standard Error (L.O.O.)	0.777	0.733	0.828	0.777	0.757	0.697	0.516	0.754
r_t_^2^ (External set)	0.525	0.285	0.302	0.010	0.541	0.708	0.439	0.690
Standard Error (External set)	0.567	0.382	0.941	0.635	0.752	0.306	0.339	0.433
Hologram lengths	151	53	353	199	61	71	59	307
Principal components	6	5	6	3	3	3	3	4
Limitation of atoms in each fragment	5–10	5–10	5–8	5–6	2–8	5–6	5–7	1–7
p-value	0.000	0.000	0.000	0.000	0.000	0.000	0.000	0.000
**Model assessment**								
δ	1.10	1.20	1.30	1.20	1.35	1.2	0.80	1.20
Assessment	+	+	+	+	+	+	+	+
Range of prediction	−1 3	−1.5 0.2	0 1.2	0.2 1	0 2.5	−0.5 1.7	0.5 2	−2 0.5
$r_{m}^{2}$	**0.765**	0.572	0.439	0.482	**0.621**	**0.500**	0.624	**0.616**
$r_{m}^{/2}$	**0.633**	0.504	0.132	0.297	**0.425**	**0.348**	0.387	**0.466**
$\bar{r_{m}^{2}}$	**0.699**	0.538	0.286	0.389	**0.523**	**0.424**	0.506	**0.541**
$\Delta r_{m}^{2}$	**0.132**	0.068	0.307	0.185	**0.196**	**0.152**	0.237	**0.150**
$r_{m(test)}^{2}$	**0.507**	0.276	0.289	0.006	**0.503**	**0.645**	0.367	**0.569**
$r_{m(test)}^{/2}$	**0.362**	0.159	−0.033	0.005	**0.342**	**0.493**	−0.054	**0.378**
$\bar{r_{m(test)}^{2}}$	**0.435**	0.217	0.128	0.005	**0.422**	**0.569**	0.154	**0.473**
$\Delta r_{m(test)}^{2}$	**0.145**	0.117	0.322	0.001	**0.161**	**0.151**	0.415	**0.190**

**Table 5:** Results of 3D-QSAR.

Model	RPMI-fs43	RPMI-fs45	CPTk5-sh44	P388-s15	CPT45-s53	KB3-s34	KB3-e12	KB3-h34	KB3-eh32	KBV-s34	KBH-fs43	TOP-I -s34	TOP-I -h54
Number compounds in training set	105	105	80	66	58	68	68	68	68	60	48	74	74
Number compounds in test set	28	28	21	16	15	15	15	15	15	21	12	20	20
R^2^ (Training set)	0.734	0.812	0.650	0.667	0.496	0.721	0.698	0.768	0.731	0.629	0.696	0.701	0.700
Standard Error (Training set)	0.601	0.510	0.522	0.537	0.589	0.597	0.593	0.528	0.559	0.523	0.394	0.535	0.536
q^2^ (L.O.O.)	0.594	0.607	0.338	0.309	0.203	0.584	0.552	0.539	0.582	0.372	0.330	0.423	0.345
Standard Error (L.O.O.)	0.742	0.737	0.718	0.774	0.741	0.707	0.722	0.744	0.697	0.680	0.586	0.743	0.792
r_t_^2^ (External set)	0.685	0.701	0.471	0.570	0.202	0.661	0.496	0.636	0.620	0.436	0.282	0.795	0.836
Standard Error (External set)	0.742	0.724	0.545	0.739	0.570	0.647	0.789	0.670	0.686	0.857	0.796	0.518	0.338
3D-descriptor	Fs	fs	sh	S	S	S	e	h	eh	s	Fs	S	H
Column filter	4	4	4	1	5	3	1	3	3	3	4	3	5
Principal component	3	5	4	5	3	4	2	4	2	4	3	4	4
p-value	0.000	0.000	0.000	0.000	0.000	0.000	0.000	0.000	0.000	0.000	0.000	0.000	0.000
**Model assessment**													
δ	1.10	1.00	1.05	1.05	1.20	1.15	1.20	1.10	1.15	1.00	0.75	1.10	1.2
Assessment	+	-	+	+	+	+	+	+	+	+/−	+	+	+
Range of prediction	−1.5 2.5	− −	−1.5 1	−0.2 2	−0.2 1	−0.5 2	0 2	−0.2 2.2	−0.2 2	0 1.2	−0.5 2	−2.5 0.5	−2.5 1
$r_{m}^{2}$	**0.571**	0.812	0.650	0.667	0.496	**0.721**	0.697	0.767	0.730	0.628	0.696	**0.701**	0.700
$r_{m}^{/2}$	**0.530**	0.703	0.592	0.442	0.312	**0.565**	0.531	0.634	0.579	0.501	0.494	**0.578**	0.577
$\bar{r_{m}^{2}}$	**0.550**	0.758	0.621	0.554	0.404	**0.643**	0.614	0.701	0.654	0.564	0.595	**0.639**	0.638
$\Delta r_{m}^{2}$	**0.041**	0.109	0.058	0.225	0.183	**0.156**	0.166	0.133	0.151	0.127	0.202	**0.123**	0.123
$r_{m(test)}^{2}$	**0.663**	0.324	0.429	0.569	0.188	**0.585**	0.461	0.636	0.591	0.332	0.282	**0.604**	0.523
$r_{m(test)}^{/2}$	**0.568**	−0.451	0.294	0.332	0.063	**0.546**	0.162	0.409	0.349	−0.014	−0.009	**0.431**	0.318
$\bar{r_{m(test)}^{2}}$	**0.616**	0.064	0.362	0.450	0.126	**0.565**	0.312	0.522	0.470	0.159	0.137	**0.517**	0.421
$\Delta r_{m(test)}^{2}$	**0.095**	0.775	0.135	0.237	0.125	**0.039**	0.299	0.278	0.242	0.346	0.291	**0.173**	0.206

**Table 6:** Chemical structure of 137 benzo[*c*]phenanthridine analogues.

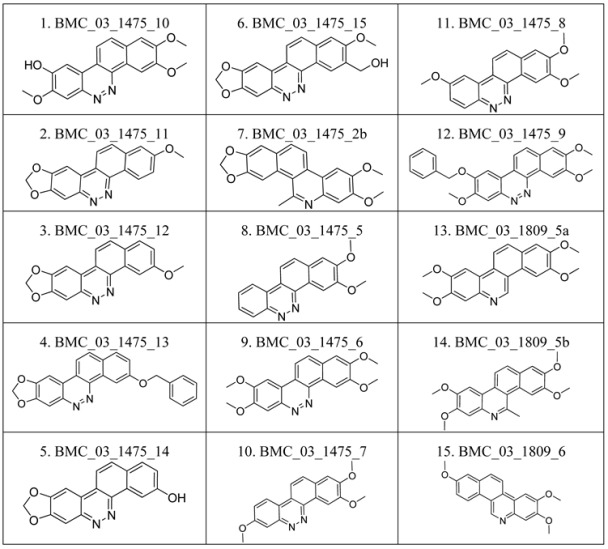 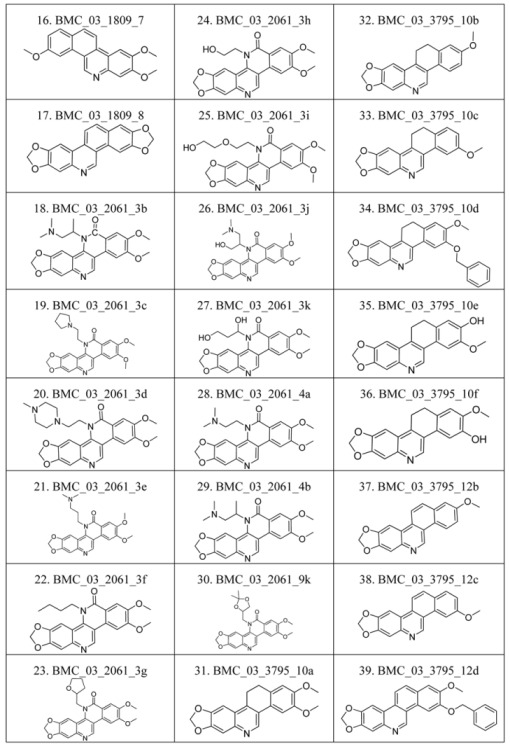 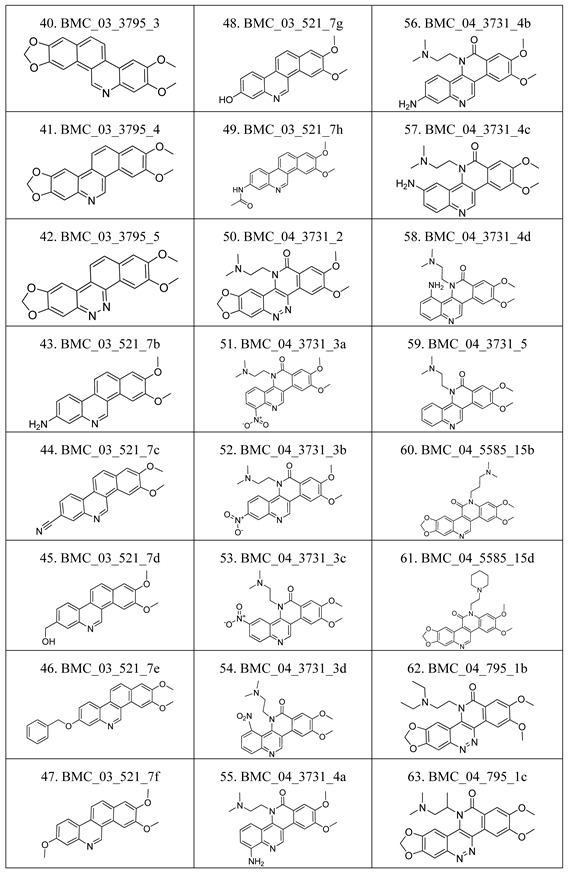 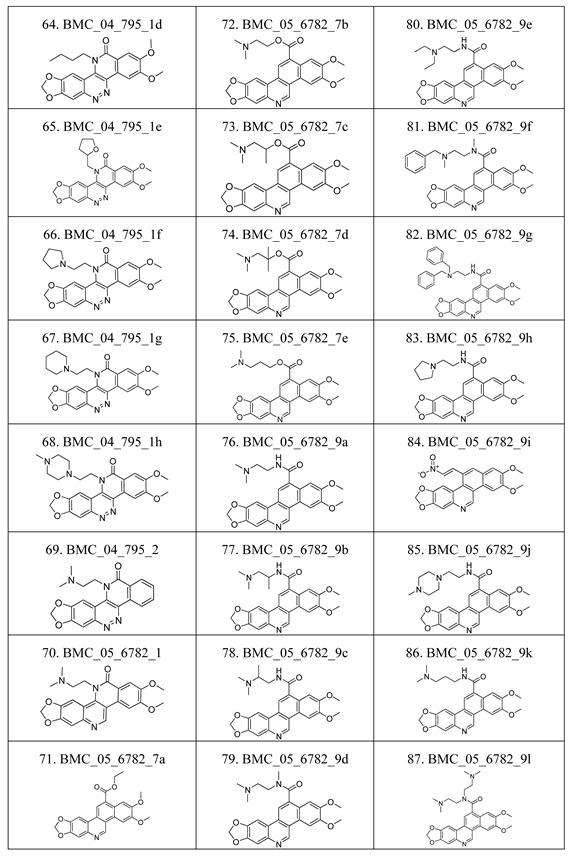 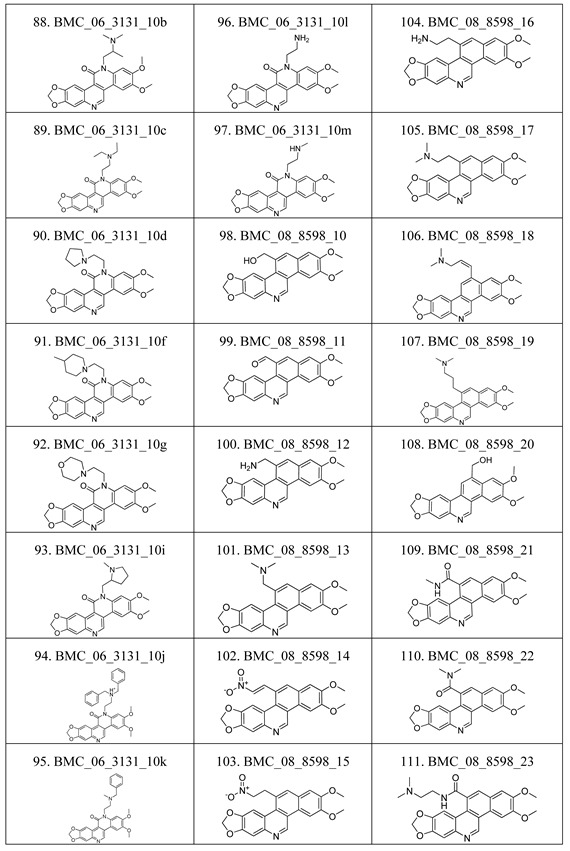 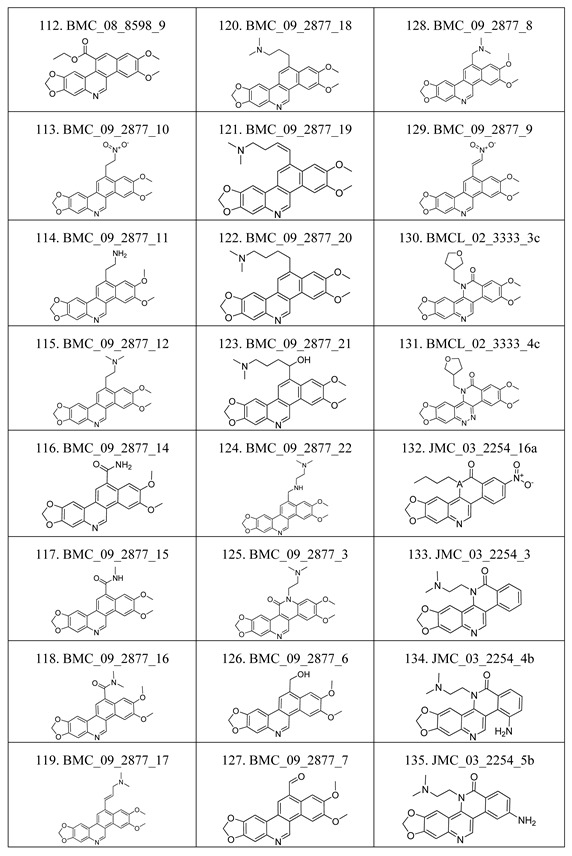 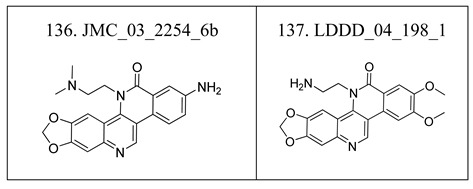

**Table 7:** Description of 35 molecular descriptors using to create the 2D QSAR models.

No	Molecular descriptor	Description
1	a_acc	Number of hydrogen bond acceptor atoms
1	a_acc	Number of hydrogen bond acceptor atoms
2	a_aro	Number of aromatic atoms
3	a_ICM	Atom information content (mean). This is the entropy of the element distribution in the molecule (including implicit hydrogens but not lone pair pseudo-atoms).
4	a_nN	Number of nitrogen atoms: #{Zi | Zi = 7}.
5	a_nO	Number of oxygen atoms: #{Zi | Zi = 8}.
6	b_1rotR	Fraction of rotatable single bonds: b_1rotN divided by b_heavy.
7	BCUT_PEOE_0	The BCUT descriptors [Pearlman 1998] are calculated from the eigenvalues of a modified adjacency matrix.
8	BCUT_PEOE_1	
9	BCUT_PEOE_2	
10	chi1v_C	Carbon valence connectivity index (order 1).
11	density	Molecular mass density: Weight divided by vdw_vol (amu/Å^3^).
12	diameter	Largest value in the distance matrix
13	GCUT_PEOE_1	The GCUT descriptors are calculated from the eigenvalues of a modified graph distance adjacency matrix.
14	GCUT_SLOGP_0	The GCUT descriptors using atomic contribution to logP instead of partial charge.
15	GCUT_SLOGP_1	
16	GCUT_SMR_0	The GCUT descriptors using atomic contribution to molar refractivity instead of partial charge.
17	opr_leadlike	Atom Counts and Bond Counts: One if and only if opr_violation < 2 otherwise zero.
18	PEOE_VSA_FHYD	Fractional hydrophobic van der Waals surface area.
19	PEOE_VSA_FNEG	Fractional negative van der Waals surface area.
20	PEOE_VSA_NEG	Total negative van der Waals surface area.
21	PEOE_VSA+0	Sum of *v_i_* where *q_i_* is in the range [0.00, 0.05).
22	PEOE_VSA+1	PEOE: Sum of *v_i_* where *q_i_* is in the range [0.05, 0.10).
23	PEOE_VSA+2	PEOE: Sum of *v_i_* where *q_i_* is in the range [0.10, 0.15).
24	PEOE_VSA+3	PEOE: Sum of *v_i_* where *q_i_* is in the range [0.15, 0.20).
25	PEOE_VSA-0	PEOE: Sum of *v_i_* where *q_i_* is in the range [−0.05, 0.00).
26	PEOE_VSA-1	PEOE: Sum of *v_i_* where *q_i_* is in the range [−0.10, −0.05).
27	petitjean	Largest value in the distance matrix
28	SlogP	Log of the octanol/water partition coefficient (including implicit hydrogens).
29	SlogP_VSA1	Subdivided Surface Areas: Sum of *v_i_* such that *L_i_* is in (−0.4, −0.2].
30	SlogP_VSA5	Subdivided Surface Areas: Sum of *v_i_* such that *L_i_* is in (0.15, 0.20].
31	SlogP_VSA9	Subdivided Surface Areas: Sum of *v_i_* such that *L_i_* > 0.40.
32	VDistMa	Adjacency and Distance Matrix Descriptors: If *m* is the sum of the distance matrix entries then VDistMa is defined to be the sum of log_2_ *m - D_ij_* log_2_ *D_ij_* / *m* over all *i* and *j*.
33	vsa_acc	Approximation to the sum of VDW surface areas (Å^2^) of pure hydrogen bond acceptors
34	vsa_other	Approximation to the sum of VDW surface areas (Å^2^) of atoms typed as “other”.
35	vsa_pol	Approximation to the sum of VDW surface areas (Å^2^) of polar atoms (atoms that are both hydrogen bond donors and acceptors), such as -OH.

**Table 8:** Cross-validation results of 3D-QSAR with KB3-1 cells.

3D descriptor field	q^2^ for each column filter values				
	1	2	3	4	5
S	0.583	0.583	0.584	0.579	0.575
E	0.552	0.551	0.553	0.540	0.542
H	0.542	0.541	0.539	0.509	0.485
D	0.014	0.013	0.004	0.000	0.000
A	0.414	0.415	0.417	0.423	0.429
s.e	0.580	0.580	0.580	0.576	0.576
s.h	0.551	0.555	0.555	0.545	0.536
s.d	0.448	0.446	0.473	0.485	0.502
s.a	0.498	0.507	0.501	0.503	0.505
e.h	0.582	0.581	0.582	0.571	0.560
e.d	0.560	0.562	0.568	0.545	0.534
e.a	0.561	0.558	0.552	0.550	0.547
h.d	0.437	0.438	0.438	0.411	0.406
h.a	0.466	0.465	0.465	0.463	0.467
d.a	0.409	0.412	0.417	0.423	0.427
s.e.h	0.585	0.581	0.584	0.579	0.570
s.e.d	0.578	0.578	0.586	0.584	0.575
s.e.a	0.563	0.564	0.562	0.561	0.558
s.h.d	0.514	0.527	0.533	0.528	0.516
s.h.a	0.511	0.515	0.511	0.510	0.511
s.d.a	0.499	0.507	0.514	0.530	0.509
e.h.d	0.563	0.565	0.573	0.560	0.552
e.h.a	0.560	0.557	0.551	0.546	0.540
e.d.a	0.526	0.528	0.533	0.531	0.520
h.d.a	0.444	0.446	0.445	0.443	0.449
s.e.h.d	0.572	0.574	0.579	0.571	0.562
s.e.h.a	0.569	0.569	0.565	0.561	0.555
s.e.d.a	0.548	0.551	0.555	0.558	0.544
s.h.d.a	0.493	0.507	0.515	0.518	0.501
e.h.d.a	0.528	0.529	0.530	0.523	0.513
s.e.h.d.a	0.545	0.547	0.543	0.540	0.537
CoMFA	0.549	0.549	0.546	0.547	0.551

s: steric, e: electrostatic, h: hydrophobic; d: H-bond donor; a: H- bond acceptor.

## Supplementary Materials

The cytotoxicity on seven tumor cell lines and toposisomerase I inhibitory activity of 137 benzo[*c*]phenanthridine analogues (eight tables) and the detailed of 29 QSAR models including hologram, 2D- and 3D- QSAR were provided. Several new designed BCPs compounds with predictive activity from QSAR models and the equations of $r_{m}^{2}$, $\bar{r_{m}^{2}}$ and $\Delta r_{m}^{2}$ metrics were also available in detailed. The detailed information can be found at: http://www.mdpi.com/1420-3049/17/5/5690/s1.
