# Supplementary material for: QSAR Modeling on Benzo[c]phenanthridine Analogues as Topoisomerase I Inhibitors and Anti-cancer Agents
Source: Molecules. 2012 May 11;17(5):5690–712. doi: 10.3390/molecules17055690 (PMC6268722; doi:10.3390/molecules17055690)
Supplement: Supplementary file 1 [file molecules-17-05690-s001.pdf]

SUPPORTING INFORMATION

QSAR Modeling on Benzo[*c*]phenanthridine Analogues as Topoisomerase I Inhibitors and Anti-cancer Agents

Thai Khac Minh,* Quang-Huynh Bui, Thanh-Dao Tran, and Thi-Ngoc-Phuong Huynh

Department of Medicinal Chemistry, School of Pharmacy, University of Medicine and Pharmacy at Ho Chi Minh City, 41 Dinh Tien Hoang, Dist. 1, Ho Chi Minh City, Vietnam

Corresponding author: Dr. Khac-Minh Thai, Tel.: +84-909-680-385; Fax: +84-8-3822-5435;
E-Mail: thaikhacminh@uphcm.edu.vn; thaikhacminh@gmail.com

Figure S1. Process of 2D-QSAR in Rapidminer software

Table S1. Dataset of 133 BCPs compounds with their cytotoxicity on RPMI8402 cell line: experimental pIC_{50} value and predictive results

Table S2. Dataset of 101 BCPs compounds with their cytotoxicity on CPTK5 cell line: experimental pIC_{50} value and predictive results

Table S3. Dataset of 82 BCPs compounds with their cytotoxicity on P388 cell line: experimental pIC_{50} value and predictive results

Table S4. Dataset of 73 BCPs compounds with their cytotoxicity on CPT45 cell line: experimental pIC_{50} value and predictive results

Table S5. Dataset of 83 BCPs compounds with their cytotoxicity on KB3-1 cell line: experimental pIC_{50} value and predictive results

Table S6. Dataset of 81 BCPs compounds with their cytotoxicity on KBV-1 cell line: experimental pIC_{50} value and predictive results

Table S7. Dataset of 60 BCPs compounds with their cytotoxicity on KBH5.0 cell line: experimental pIC_{50} value and predictive results

Table S8. Dataset of 94 BCPs compounds with experimental topoisomerase-1 inhibitory value and predictive results

Model 1. 2D QSAR model on RPMI8402 cytotoxicity

Model 2. 2D QSAR model on CPTK5 cytotoxicity

Model 3. 2D QSAR model on P388 cytotoxicity

Model 4. 2D QSAR model on CPT45 cytotoxicity

Model 5. 2D QSAR model on KB3-1 cytotoxicity

Model 6. 2D QSAR model on KBV-1 cytotoxicity

Model 7. 2D QSAR model on KBH5.0 cytotoxicity

Model 8. 2D QSAR model on topoisomerase-1 inhibitory activity

Model 9. Hologram QSAR model on RPMI8402 cytotoxicity

Model 10. Hologram QSAR model on CPTK5 cytotoxicity

Model 11. Hologram QSAR model on P388 cytotoxicity

Model 12. Hologram QSAR model on CPT45 cytotoxicity

Model 13. Hologram QSAR model on KB3-1 cytotoxicity

Model 14. QSAR model on KBV-1 cytotoxicity
Model 15. Hologram QSAR model on KBH5.0 cytotoxicity
Model 16. Hologram QSAR model on topoisomerase-1 inhibitory activity
Model 17-18. 3D QSAR model on RPMI8402 cytotoxicity
Model 19. 3D QSAR model on CPTK5 cytotoxicity
Model 20. 3D QSAR model on P388 cytotoxicity
Model 21. 3D QSAR model on CPT45 cytotoxicity
Model 22-25. 3D QSAR model on KB3-1 cytotoxicity
Model 26. 3D QSAR model on KBV-1 cytotoxicity
Model 27. 3D QSAR model on KBH5.0 cytotoxicity
Model 28-29. 3D QSAR model on topoisomerase-1 inhibitory activity
Table S9. Several new designed BCPs compound with predictive activity from QSAR models
Part S. Calculation of r_m^2 metrics and references.

Figure S1. Process of 2D-QSAR in Rapidminer software

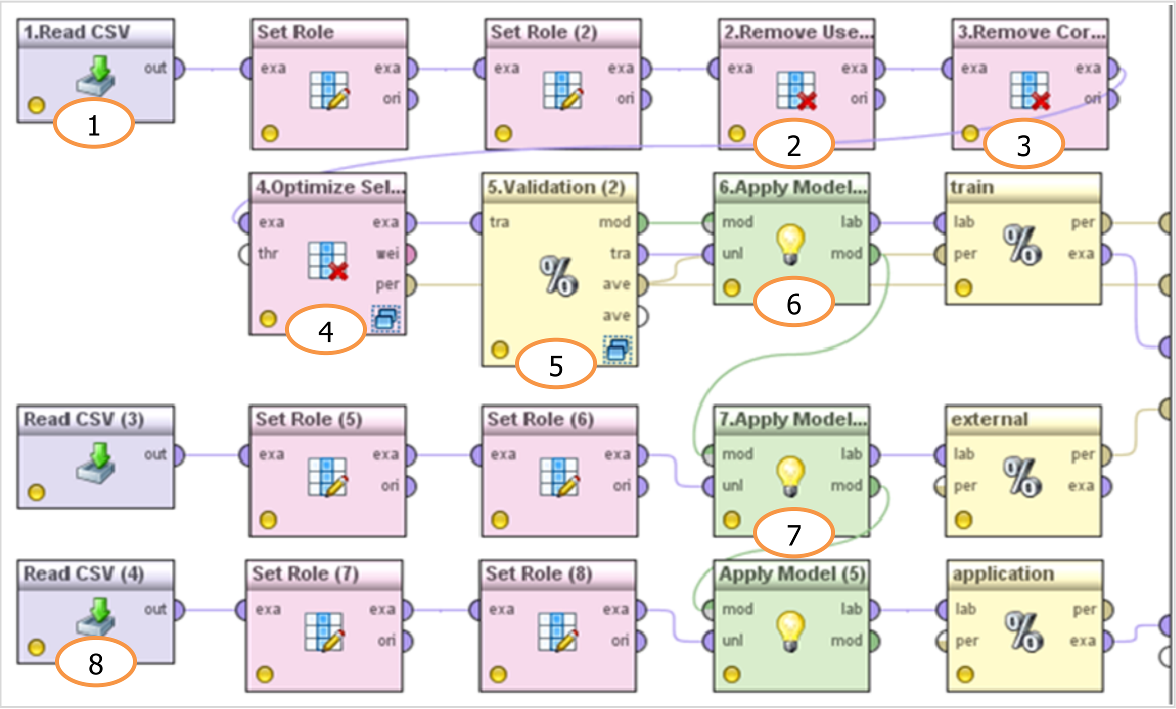

Table S1. Dataset of 133 BCPs compounds with their cytotoxicity on RPMI8402 cell line: experimental pIC_{50} value and predictive results

No	Name	pIC50/RPMI	pIC50/RPMI-S43	pIC50/RPMI-S45	pIC50/RPMI-151	pIC50/RPMI-2D
1	BMCL_02_3333_4C	2.00	2.10	2.07	1.74	1.41
2	BMC_03_1475_12	0.35	-0.11	-0.06	-0.22	0.43
3	BMC_03_1475_14	-1.00	-0.21	-0.61	-0.16	0.17
4	BMC_03_1475_15	0.49	0.26	0.38	0.00	0.52
5	BMC_03_1475_2B	-0.88	-1.66	-0.85	-1.07	-0.13
6	BMC_03_1475_5	-0.74	-0.40	-0.68	-0.74	-0.50
7	BMC_03_1475_8	-0.60	0.21	-0.06	-0.92	-0.45
8	BMC_03_1475_9	-1.59	-1.25	-0.89	-1.23	-0.86
9	BMC_03_1809_5A	-1.15	-1.20	-0.80	-0.81	-0.54
10	BMC_03_1809_5B	-1.34	-1.26	-0.74	-1.80	-0.61
11	BMC_03_1809_6	-0.70	-1.54	-1.01	-0.39	-0.88
12	BMC_03_2061_3C	2.52	1.53	1.56	1.91	1.96
13	BMC_03_2061_3D	0.47	1.39	1.14	1.39	1.54
14	BMC_03_2061_3F	1.10	1.45	1.51	1.71	1.16
15	BMC_03_2061_3G	0.82	1.53	1.44	1.54	1.04
16	BMC_03_2061_3H	1.70	1.25	1.25	1.52	1.22
17	BMC_03_2061_3I	1.59	1.39	1.73	1.65	1.49
18	BMC_03_2061_3J	-0.20	0.30	-0.27	-0.18	1.53
19	BMC_03_2061_3K	1.36	1.29	1.20	1.30	1.28
20	BMC_03_2061_4A	1.77	1.44	1.58	1.65	1.38
21	BMC_03_2061_4B	0.80	0.97	0.94	0.16	1.27
22	BMC_03_2061_9K	1.41	1.75	1.64	1.26	1.49
23	BMC_03_3795_10A	0.40	0.13	-0.30	0.16	0.37
24	BMC_03_3795_10C	-1.52	-0.47	-1.01	-0.56	0.40
25	BMC_03_3795_10D	0.80	0.38	0.27	0.40	0.01
26	BMC_03_3795_10E	-0.88	0.10	-0.47	0.05	0.74
27	BMC_03_3795_12B	0.70	-0.01	-0.06	0.63	-0.70
28	BMC_03_3795_12D	0.52	0.77	0.99	0.23	-0.47
29	BMC_03_3795_3	-0.65	-1.67	-0.97	-0.11	-0.14
30	BMC_03_3795_5	1.15	0.37	0.19	-0.21	0.48
31	BMC_03_521_7B	-0.36	-0.26	-0.45	-0.72	-0.93
32	BMC_03_521_7C	-0.90	-0.56	-0.70	-0.93	-0.72
33	BMC_03_521_7D	-1.11	-0.44	-0.58	-0.78	-0.85
34	BMC_03_521_7E	-0.93	-0.96	-0.94	-0.66	-1.59
35	BMC_03_521_7G	-0.20	-0.40	-0.60	-0.60	-1.08
36	BMC_03_521_7H	-0.60	-0.62	-0.68	-0.43	-0.21
37	BMC_04_3731_2	3.00	2.41	2.53	2.08	1.64
38	BMC_04_3731_3A	0.66	0.59	0.59	0.96	0.75
39	BMC_04_3731_3B	1.12	0.82	0.93	0.90	1.13
40	BMC_04_3731_4A	0.19	0.42	0.44	0.73	0.59
41	BMC_04_3731_4B	1.00	0.82	0.96	0.88	0.24
42	BMC_04_3731_5	1.00	0.64	0.66	0.69	0.39
43	BMC_04_5585_15B	3.30	1.76	2.32	2.44	2.15
44	BMC_04_5585_15D	3.00	2.58	2.82	2.61	1.88

No	Name	pIC50/RPMI	pIC50/RPMI-S43	pIC50/RPMI-S45	pIC50/RPMI-151	pIC50/RPMI-2D
45	BMC_04_795_1B	2.40	2.38	2.60	2.25	2.27
46	BMC_04_795_1C	0.59	0.54	0.08	0.90	1.55
47	BMC_04_795_1D	2.40	2.13	2.12	2.06	1.54
48	BMC_04_795_1E	2.00	1.09	1.31	2.02	1.38
49	BMC_04_795_1F	2.52	2.34	2.33	2.43	2.17
50	BMC_04_795_1G	2.52	2.38	2.33	2.40	2.11
51	BMC_04_795_1H	1.10	2.20	1.94	1.91	1.70
52	BMC_04_795_2	0.62	1.31	1.06	1.10	1.22
53	BMC_05_6782_7A	1.07	1.22	1.17	1.31	0.80
54	BMC_05_6782_7B	1.52	1.35	1.68	1.38	1.32
55	BMC_05_6782_7C	1.52	1.57	1.74	1.39	1.34
56	BMC_05_6782_7D	1.40	1.15	1.31	1.26	1.38
57	BMC_05_6782_7E	0.66	0.62	0.42	1.42	1.00
58	BMC_05_6782_9C	2.70	1.95	2.27	1.40	2.12
59	BMC_05_6782_9D	0.30	0.56	0.17	1.36	1.41
60	BMC_05_6782_9E	2.22	1.61	1.66	1.39	1.68
61	BMC_05_6782_9F	1.47	0.78	1.07	0.97	0.84
62	BMC_05_6782_9G	0.35	0.83	0.04	0.50	0.94
63	BMC_05_6782_9H	2.52	1.62	1.79	1.52	1.36
64	BMC_05_6782_9I	1.26	1.56	1.57	1.47	2.05
65	BMC_05_6782_9J	1.19	1.25	1.05	0.99	1.94
66	BMC_05_6782_9K	1.30	1.58	1.57	1.21	1.63
67	BMC_05_6782_9L	0.77	0.49	0.28	1.62	1.21
68	BMC_06_3131_10B	2.52	2.19	2.63	2.31	2.27
69	BMC_06_3131_10C	2.52	2.61	2.94	2.50	2.14
70	BMC_06_3131_10F	2.40	2.43	2.46	2.72	1.44
71	BMC_06_3131_10G	1.46	2.46	2.61	2.45	1.96
72	BMC_06_3131_10I	1.52	1.77	1.95	2.23	1.94
73	BMC_06_3131_10J	1.30	0.95	2.02	1.26	0.71
74	BMC_06_3131_10K	1.64	2.14	1.72	1.99	1.29
75	BMC_06_3131_10L	2.70	1.85	2.04	2.39	1.52
76	BMC_06_3131_10M	2.70	2.31	2.72	2.40	2.26
77	BMC_08_8598_10	0.92	1.03	0.93	0.81	0.07
78	BMC_08_8598_12	0.92	1.04	0.98	0.87	0.60
79	BMC_08_8598_13	1.00	0.59	0.57	1.30	1.06
80	BMC_08_8598_14	0.26	1.13	0.86	0.01	0.42
81	BMC_08_8598_15	1.22	1.24	1.35	1.77	0.51
82	BMC_08_8598_17	1.70	1.70	1.78	1.65	1.06
83	BMC_08_8598_19	1.22	0.67	1.05	1.53	1.34
84	BMC_08_8598_21	0.55	0.67	0.51	0.26	1.04
85	BMC_08_8598_23	1.46	0.87	1.26	0.58	2.12
86	BMC_08_8598_9	-1.00	0.53	0.19	-0.66	0.14
87	BMC_09_2877_10	0.42	1.29	1.26	1.05	1.23
88	BMC_09_2877_11	1.60	1.33	1.39	0.90	0.73
89	BMC_09_2877_12	1.52	1.72	1.99	1.00	1.45
90	BMC_09_2877_14	-0.60	0.96	0.67	1.10	1.30

No	Name	pIC50/RPMI	pIC50/RPMI-S43	pIC50/RPMI-S45	pIC50/RPMI-151	pIC50/RPMI-2D
91	BMC_09_2877_16	0.22	0.77	0.58	1.01	0.71
92	BMC_09_2877_17	1.35	1.50	0.99	1.54	1.39
93	BMC_09_2877_18	0.74	1.38	1.69	0.83	1.43
94	BMC_09_2877_19	0.52	1.19	0.28	1.40	0.88
95	BMC_09_2877_20	0.00	1.17	1.05	0.90	1.00
96	BMC_09_2877_21	0.52	1.16	0.37	0.86	1.16
97	BMC_09_2877_22	1.62	1.18	1.14	1.49	1.32
98	BMC_09_2877_3	3.15	2.48	2.88	2.45	2.24
99	BMC_09_2877_7	2.52	0.91	0.95	1.13	0.64
100	BMC_09_2877_8	1.48	1.42	1.31	1.04	0.80
101	BMC_09_2877_9	1.48	1.25	1.09	1.17	1.07
102	JMC_03_2254_16A	0.62	0.84	0.91	0.63	1.45
103	JMC_03_2254_3	1.22	0.80	0.65	0.62	0.91
104	JMC_03_2254_4B	0.47	0.71	0.47	0.62	1.15
105	JMC_03_2254_6B	1.10	0.89	1.06	0.83	1.55
106*	BMCL_02_3333_3C	0.82	1.64	3.34	1.45	1.06
107*	BMC_03_1475_11	-1.61	0.02	1.53	-0.03	0.04
108*	BMC_03_1475_7	-0.30	0.27	1.62	-0.83	-0.17
109*	BMC_03_1809_7	-0.70	-1.68	1.03	-0.70	-1.22
110*	BMC_03_2061_3B	0.24	0.31	2.14	0.16	1.27
111*	BMC_03_2061_3E	1.52	1.56	3.05	1.68	2.03
112*	BMC_03_3795_10B	0.22	-0.23	1.05	-0.01	0.00
113*	BMC_03_3795_10F	-0.15	-0.28	0.74	0.11	0.34
114*	BMC_03_3795_12C	-1.26	-0.15	1.62	0.31	-0.30
115*	BMC_03_3795_4	0.40	0.34	1.65	0.20	-0.19
116*	BMC_03_521_7F	-0.59	-0.88	0.92	-0.51	-0.86
117*	BMC_04_3731_3C	1.74	0.84	2.88	1.01	0.78
118*	BMC_04_3731_3D	-0.74	-0.76	0.65	0.78	1.16
119*	BMC_04_3731_4C	1.40	0.75	2.85	0.84	0.61
120*	BMC_04_3731_4D	-0.60	0.27	2.24	0.95	0.60
121*	BMC_05_6782_1	2.70	1.44	3.32	1.65	1.38
122*	BMC_05_6782_9A	2.52	1.83	3.86	1.40	2.06
123*	BMC_05_6782_9B	3.40	1.80	3.65	1.56	1.80
124*	BMC_06_3131_10D	2.52	2.48	4.17	2.63	1.91
125*	BMC_08_8598_11	1.00	0.90	2.39	0.00	0.67
126*	BMC_08_8598_16	1.49	1.25	3.10	1.08	0.78
127*	BMC_08_8598_18	1.46	1.07	2.69	0.11	1.39
128*	BMC_08_8598_20	0.85	0.72	2.50	0.17	1.32
129*	BMC_08_8598_22	-0.20	0.61	2.53	0.35	0.74
130*	BMC_09_2877_15	1.40	1.36	3.37	1.03	1.03
131*	BMC_09_2877_6	1.52	1.11	2.97	0.92	0.05
132*	JMC_03_2254_5B	1.60	1.04	3.12	0.78	1.18
133*	LDDD_04_198_1	2.40	1.23	2.86	1.54	1.52

*: external test set

Table S2. Dataset of 101 BCPs compounds with their cytotoxicity on CPTK5 cell line: experimental pIC_{50} value and predictive results

No	Name	pIC50/CPTk5	pIC50/CPTk5-SH44	pIC50/CPTk5-53	pIC50/CPTk5-2D
1	BMC_03_1475_12	-2.00	-1.48	-0.83	-0.47
2	BMC_03_1475_15	0.42	-0.54	-0.41	-0.60
3	BMC_03_1475_2B	-0.99	-1.60	-0.56	0.08
4	BMC_03_1475_5	-1.00	-0.88	-1.54	-1.39
5	BMC_03_1475_8	-1.45	-0.52	-1.36	-1.18
6	BMC_03_1475_9	-1.79	-1.95	-1.59	-1.02
7	BMC_03_1809_5A	-1.20	-1.04	-0.55	-0.64
8	BMC_03_1809_5B	-1.04	-0.98	-0.99	-0.64
9	BMC_03_1809_6	-0.88	-0.99	-0.38	-0.99
10	BMC_03_2061_3C	0.41	-0.09	-0.11	-0.05
11	BMC_03_2061_3D	-1.00	-0.36	-0.36	-0.46
12	BMC_03_2061_3J	-1.10	-0.76	-0.68	-0.36
13	BMC_03_2061_4A	-0.60	0.03	-0.17	0.11
14	BMC_03_2061_4B	0.40	0.09	-0.36	0.18
15	BMC_03_3795_10A	0.70	-0.21	-0.21	-0.22
16	BMC_03_3795_10C	-1.85	-1.26	-0.86	-0.57
17	BMC_03_3795_10D	0.85	0.84	-0.09	-0.41
18	BMC_03_3795_10E	-1.40	-1.01	-0.76	-0.90
19	BMC_03_3795_12B	0.70	-0.05	0.29	-0.27
20	BMC_03_3795_12D	0.52	0.86	0.14	-0.11
21	BMC_03_3795_3	-1.04	-1.71	-0.14	0.08
22	BMC_03_3795_5	-0.74	-0.47	-0.92	-0.12
23	BMC_03_521_7B	-0.75	-0.97	-1.67	-0.56
24	BMC_03_521_7D	-1.60	-0.90	-1.36	-1.39
25	BMC_03_521_7E	-1.36	-1.34	-0.83	-1.18
26	BMC_03_521_7G	-1.15	-0.66	-1.19	-1.67
27	BMC_03_521_7H	-1.48	-1.42	-1.54	-0.72
28	BMC_04_3731_2	0.22	0.07	-0.35	-0.02
29	BMC_04_3731_3A	-0.48	-0.42	-0.12	-0.76
30	BMC_04_3731_3B	-0.53	-0.49	0.17	-0.81
31	BMC_04_3731_4B	-0.32	-0.82	0.09	-0.62
32	BMC_04_3731_5	-0.23	-0.37	0.05	-1.25
33	BMC_04_5585_15B	0.70	0.54	0.51	0.09
34	BMC_04_5585_15D	-0.18	-0.02	0.20	0.22
35	BMC_04_795_1B	0.29	0.01	-0.40	0.14
36	BMC_04_795_1C	-0.56	-1.03	-0.61	0.04
37	BMC_04_795_1D	-1.00	-0.36	-0.54	0.24
38	BMC_04_795_1F	0.22	-0.04	-0.29	-0.18
39	BMC_04_795_1G	-0.04	-0.11	-0.29	0.02
40	BMC_04_795_1H	-0.90	-0.25	-0.56	-0.59
41	BMC_04_795_2	-0.40	-0.52	-1.30	-0.65
42	BMC_05_6782_7B	0.14	0.23	0.14	-0.15
43	BMC_05_6782_7C	0.00	0.13	0.03	-0.39

No	Name	pIC50/CPTk5	pIC50/CPTk5-SH44	pIC50/CPTk5-53	pIC50/CPTk5-2D
44	BMC_05_6782_7D	-0.35	-0.04	-0.19	-0.43
45	BMC_05_6782_7E	-0.18	0.17	0.14	0.01
46	BMC_05_6782_9C	0.12	-0.07	-0.13	0.23
47	BMC_05_6782_9D	-0.70	-0.47	-0.55	-0.23
48	BMC_05_6782_9E	-0.24	-0.25	-0.37	-0.01
49	BMC_05_6782_9F	-1.00	-0.88	-0.77	-0.73
50	BMC_05_6782_9H	-0.34	-0.15	-0.28	-0.33
51	BMC_05_6782_9I	-0.81	-0.33	-0.30	-0.13
52	BMC_05_6782_9J	-0.70	-0.27	-0.49	-0.74
53	BMC_05_6782_9K	-0.32	-0.11	-0.47	-0.02
54	BMC_05_6782_9L	-0.30	-0.49	-0.76	-0.11
55	BMC_06_3131_10B	0.74	0.51	0.79	0.33
56	BMC_06_3131_10C	0.05	0.24	0.14	0.34
57	BMC_06_3131_10F	0.40	-0.25	0.09	0.27
58	BMC_06_3131_10I	0.52	0.55	0.68	0.09
59	BMC_06_3131_10J	-0.32	-0.93	-0.01	-0.23
60	BMC_06_3131_10K	-0.28	-0.42	0.11	-0.32
61	BMC_06_3131_10L	0.52	0.47	0.35	0.27
62	BMC_06_3131_10M	0.40	0.33	0.45	0.20
63	BMC_08_8598_12	-0.32	0.42	-0.38	0.38
64	BMC_08_8598_13	-0.43	0.26	-0.28	0.99
65	BMC_08_8598_17	0.48	0.79	0.61	0.55
66	BMC_08_8598_19	0.54	0.23	0.20	0.80
67	BMC_08_8598_23	0.20	0.52	0.52	-0.18
68	BMC_09_2877_10	0.52	0.65	1.06	-0.03
69	BMC_09_2877_11	0.60	0.70	0.62	-0.14
70	BMC_09_2877_12	0.35	0.73	0.47	-0.22
71	BMC_09_2877_17	1.08	0.54	0.72	1.27
72	BMC_09_2877_18	0.85	0.71	0.58	-0.06
73	BMC_09_2877_19	0.66	0.68	0.89	0.63
74	BMC_09_2877_20	-0.36	0.54	0.51	0.10
75	BMC_09_2877_21	-0.32	0.41	0.48	-0.32
76	BMC_09_2877_22	1.52	0.65	0.97	0.10
77	BMC_09_2877_3	0.68	0.46	0.45	0.17
78	BMC_09_2877_7	2.52	0.16	-0.07	0.89
79	BMC_09_2877_8	1.48	1.23	0.90	1.16
80	BMC_09_2877_9	1.46	0.80	0.81	0.86
81	BMC_03_1475_11*	-1.61	-0.43	-0.92	-0.47
82	BMC_03_1475_7*	-0.70	-0.95	-1.40	-1.18
83	BMC_03_1809_7*	-0.93	-2.04	-0.49	-0.99
84	BMC_03_2061_3B*	-0.53	-0.86	-0.36	0.18
85	BMC_03_2061_3E*	0.05	-0.06	-0.08	0.02
86	BMC_03_3795_10B*	0.10	-0.75	-0.41	-0.57
87	BMC_03_3795_10F*	-0.04	-0.26	-0.37	-0.90
88	BMC_03_3795_4*	0.40	-0.10	0.11	0.08
89	BMC_03_521_7F*	-1.40	-1.03	-0.97	-0.99

No	Name	pIC50/CPTk5	pIC50/CPTk5-SH44	pIC50/CPTk5-53	pIC50/CPTk5-2D
90	BMC_04_3731_3C*	0.10	-0.39	-0.19	-0.81
91	BMC_04_3731_3D*	-1.08	-1.29	0.20	-0.73
92	BMC_04_3731_4A*	-0.30	-0.63	-0.14	-0.62
93	BMC_04_3731_4C*	-0.15	-0.29	-0.09	-0.62
94	BMC_04_3731_4D*	-0.90	-0.41	0.23	-0.62
95	BMC_05_6782_1*	0.05	0.03	-0.17	0.11
96	BMC_05_6782_9A*	0.00	0.13	-0.36	-0.18
97	BMC_05_6782_9B*	-0.48	-0.15	-0.48	0.13
98	BMC_06_3131_10D*	0.07	0.16	0.19	0.02
99	BMC_08_8598_16*	0.70	0.81	0.21	-0.14
100	BMC_08_8598_18*	0.46	0.37	-0.53	1.27
101	BMC_09_2877_6*	1.52	0.21	0.31	-0.05

*: external test set

Table S3. Dataset of 82 BCPs compounds with their cytotoxicity on P388 cell line: experimental pIC_{50} value and predictive results

STT	Tên	pIC50/P388	pIC50/P388-S15	pIC50/P388-353	pIC50/P388-2D
1	BMC_03_3795_10A	0.72	0.31	0.30	0.70
2	BMC_03_3795_10C	-0.65	-0.36	-0.04	-0.20
3	BMC_03_3795_10E	-0.38	0.05	0.16	-0.23
4	BMC_03_3795_12B	0.54	0.25	0.58	0.16
5	BMC_03_3795_12D	1.00	0.96	0.92	0.53
6	BMC_03_3795_3	0.35	-0.17	0.93	1.08
7	BMC_03_3795_5	0.48	0.73	0.73	1.17
8	BMC_04_3731_2	2.70	2.29	1.84	1.87
9	BMC_04_3731_3A	0.72	0.94	0.75	0.38
10	BMC_04_3731_3B	1.52	1.25	0.77	1.44
11	BMC_04_3731_3C	1.40	1.25	0.74	0.60
12	BMC_04_3731_3D	-0.78	-0.61	0.63	0.40
13	BMC_04_3731_4A	0.46	0.88	0.58	0.60
14	BMC_04_3731_4B	1.22	1.29	0.64	1.38
15	BMC_04_3731_5	1.15	1.08	0.57	0.82
16	BMC_04_5585_15B	2.52	1.95	2.89	2.83
17	BMC_04_5585_15D	2.52	2.95	3.09	2.57
18	BMC_04_795_1B	2.70	2.53	1.91	2.46
19	BMC_04_795_1C	0.96	0.75	1.91	1.80
20	BMC_04_795_1D	2.40	2.11	1.83	1.69
21	BMC_04_795_1E	1.70	1.93	1.92	2.12
22	BMC_04_795_1F	2.52	2.28	2.03	2.45
23	BMC_04_795_1G	2.52	2.30	2.05	2.20
24	BMC_04_795_1H	1.10	2.03	1.94	1.43
25	BMC_04_795_2	1.05	1.15	1.33	1.05
26	BMC_05_6782_7A	1.40	1.21	1.28	1.03
27	BMC_05_6782_7B	1.85	1.67	1.30	1.02
28	BMC_05_6782_7C	1.70	2.08	1.37	1.14

STT	Tên	pIC50/P388	pIC50/P388-S15	pIC50/P388-353	pIC50/P388-2D
29	BMC_05_6782_7D	1.40	1.54	1.46	1.56
30	BMC_05_6782_7E	0.57	0.49	1.30	1.11
31	BMC_05_6782_9C	2.70	2.28	1.42	1.66
32	BMC_05_6782_9D	0.82	0.94	1.39	1.04
33	BMC_05_6782_9E	2.40	1.90	1.45	2.04
34	BMC_05_6782_9F	1.46	1.11	1.23	1.54
35	BMC_05_6782_9G	0.46	0.23	1.10	0.86
36	BMC_05_6782_9H	2.52	1.85	1.56	1.93
37	BMC_05_6782_9I	2.00	1.83	1.58	2.32
38	BMC_05_6782_9J	1.35	1.51	1.47	1.53
39	BMC_05_6782_9K	1.52	2.12	1.37	2.05
40	BMC_05_6782_9L	0.89	0.70	1.47	1.17
41	BMC_06_3131_10C	2.52	2.71	2.95	2.78
42	BMC_08_8598_10	1.22	1.19	1.26	0.52
43	BMC_08_8598_12	1.59	1.31	1.32	1.75
44	BMC_08_8598_13	1.00	1.10	1.55	1.55
45	BMC_08_8598_14	0.74	1.45	1.11	0.62
46	BMC_08_8598_15	1.15	1.57	1.24	0.84
47	BMC_08_8598_17	1.82	1.83	1.51	1.54
48	BMC_08_8598_19	1.40	1.06	1.30	1.31
49	BMC_08_8598_21	1.60	1.08	1.27	1.15
50	BMC_08_8598_23	1.82	1.38	1.36	1.52
51	BMC_08_8598_9	-0.60	0.87	1.08	0.42
52	BMC_09_2877_10	0.55	1.20	1.06	1.44
53	BMC_09_2877_11	1.80	1.22	1.10	1.86
54	BMC_09_2877_12	1.52	1.78	1.18	0.82
55	BMC_09_2877_14	-0.20	0.98	1.29	0.34
56	BMC_09_2877_16	0.32	0.90	1.32	0.62
57	BMC_09_2877_17	1.52	1.94	1.28	2.25
58	BMC_09_2877_18	1.42	1.62	1.09	0.91
59	BMC_09_2877_19	1.52	1.17	1.33	0.85
60	BMC_09_2877_20	-0.15	1.44	1.11	1.05
61	BMC_09_2877_21	0.39	0.13	1.16	0.64
62	BMC_09_2877_22	1.70	1.52	1.20	1.33
63	BMC_09_2877_3	2.70	2.30	2.86	2.83
64	BMC_09_2877_7	2.70	0.99	1.20	2.48
65	BMC_09_2877_8	2.30	1.82	1.26	1.50
66	BMC_09_2877_9	1.60	1.39	1.30	1.23
67	BMC_03_3795_10B*	0.80	-0.05	0.05	0.04
68	BMC_03_3795_10F*	0.44	0.01	0.18	0.01
69	BMC_03_3795_12C*	-0.42	-0.15	0.62	-0.08
70	BMC_03_3795_4*	0.64	0.68	0.91	1.08
71	BMC_04_3731_4C*	1.70	1.22	0.63	1.14
72	BMC_04_3731_4D*	-0.48	0.91	0.74	0.87
73	BMC_05_6782_1*	3.00	1.44	1.25	2.24
74	BMC_05_6782_9A*	2.52	2.00	1.39	1.58

STT	Tên	pIC50/P388	pIC50/P388-S15	pIC50/P388-353	pIC50/P388-2D
75	BMC_05_6782_9B*	3.15	2.38	1.45	1.66
76	BMC_08_8598_11*	0.92	0.97	1.08	2.50
77	BMC_08_8598_16*	1.85	1.53	1.28	1.60
78	BMC_08_8598_18*	1.40	1.87	1.10	2.19
79	BMC_08_8598_20*	1.46	0.99	1.24	0.35
80	BMC_08_8598_22*	0.01	1.04	1.30	0.59
81	BMC_09_2877_15*	1.52	1.17	1.31	1.16
82	BMC_09_2877_6*	1.52	1.13	1.07	0.52

*: external test set

Table S4. Dataset of 73 BCPs compounds with their cytotoxicity on CPT45 cell line: experimental pIC_{50} value and predictive results

No	Name	pIC50/CPT45	pIC50/CPT45-S53	pIC50/CPT45-199	pIC50/CPT45-2D
1	BMC_03_3795_10A	0.74	-0.03	-0.90	0.20
2	BMC_03_3795_10C	-1.34	-0.31	-0.94	-0.47
3	BMC_03_3795_10E	-1.28	-0.23	-1.02	-0.70
4	BMC_03_3795_12B	0.28	0.19	0.63	0.49
5	BMC_03_3795_12D	0.74	0.61	0.52	0.40
6	BMC_03_3795_3	0.34	-0.05	0.81	0.80
7	BMC_04_3731_2	0.44	0.49	0.58	0.91
8	BMC_04_3731_3A	-0.32	0.30	-0.11	0.12
9	BMC_04_3731_3B	0.47	0.32	0.38	0.08
10	BMC_04_3731_3D	-0.85	-0.97	-0.03	-0.24
11	BMC_04_3731_4A	0.68	0.19	0.32	0.10
12	BMC_04_3731_4B	0.64	0.34	0.23	0.13
13	BMC_04_3731_5	0.52	0.31	0.26	0.41
14	BMC_04_5585_15B	1.70	1.03	1.56	1.37
15	BMC_04_5585_15D	0.64	0.94	1.24	0.15
16	BMC_04_795_1B	0.55	0.40	0.45	0.40
17	BMC_04_795_1C	0.25	-0.39	0.58	0.74
18	BMC_04_795_1D	-0.08	0.42	0.62	0.17
19	BMC_04_795_1E	-0.26	-0.22	0.50	-0.10
20	BMC_04_795_1F	0.59	0.40	0.41	0.30
21	BMC_04_795_1G	0.57	0.35	0.30	-0.01
22	BMC_04_795_1H	-0.04	0.18	-0.09	0.34
23	BMC_04_795_2	0.96	0.16	0.53	0.85
24	BMC_05_6782_7A	-0.51	0.94	0.92	0.28
25	BMC_05_6782_7B	1.48	1.23	1.02	0.74
26	BMC_05_6782_7C	1.70	1.31	1.04	0.40
27	BMC_05_6782_7D	0.89	0.88	1.07	-0.16
28	BMC_05_6782_7E	0.60	0.62	0.96	0.39
29	BMC_05_6782_9C	0.27	0.57	0.52	0.85
30	BMC_05_6782_9D	0.52	0.01	0.38	0.53
31	BMC_05_6782_9E	0.43	0.12	0.41	0.58
32	BMC_05_6782_9F	-0.44	-0.05	-0.39	0.51

No	Name	pIC50/CPT45	pIC50/CPT45-S53	pIC50/CPT45-199	pIC50/CPT45-2D
33	BMC_05_6782_9G	-0.81	-0.66	-1.00	-0.18
34	BMC_05_6782_9H	0.48	0.25	0.37	-0.11
35	BMC_05_6782_9I	0.00	0.02	0.26	-0.47
36	BMC_05_6782_9J	-0.13	-0.02	-0.13	0.37
37	BMC_05_6782_9K	0.47	0.50	0.67	1.06
38	BMC_05_6782_9L	-0.42	-0.84	0.09	0.30
39	BMC_06_3131_10C	1.52	1.32	1.38	0.99
40	BMC_08_8598_12	0.52	0.59	0.73	1.07
41	BMC_08_8598_13	-0.30	0.47	0.12	0.92
42	BMC_08_8598_14	-1.00	0.28	0.49	0.33
43	BMC_08_8598_17	0.74	0.88	0.16	0.28
44	BMC_08_8598_19	0.70	0.74	0.71	0.23
45	BMC_08_8598_23	0.59	0.56	0.26	0.98
46	BMC_09_2877_10	0.54	0.91	0.73	0.35
47	BMC_09_2877_11	0.52	0.98	0.75	0.40
48	BMC_09_2877_12	0.52	1.15	0.71	0.46
49	BMC_09_2877_17	1.43	1.17	1.03	1.19
50	BMC_09_2877_18	1.00	0.91	0.70	0.16
51	BMC_09_2877_19	1.22	1.09	0.94	1.48
52	BMC_09_2877_20	-0.32	0.64	0.63	-0.17
53	BMC_09_2877_21	0.29	1.22	0.58	0.48
54	BMC_09_2877_22	1.62	0.73	0.76	1.84
55	BMC_09_2877_3	1.82	1.42	1.51	1.51
56	BMC_09_2877_7	2.70	0.74	0.90	1.67
57	BMC_09_2877_8	1.82	1.68	1.10	1.76
58	BMC_09_2877_9	1.60	1.27	0.82	0.57
59	BMC_03_3795_10B*	0.60	-0.28	-0.92	-0.48
60	BMC_03_3795_10F*	0.05	-0.11	-1.04	-0.49
61	BMC_03_3795_12C*	-0.42	0.04	0.67	0.54
62	BMC_03_3795_4*	0.59	0.42	0.81	1.03
63	BMC_04_3731_3C*	0.70	0.30	0.36	-0.07
64	BMC_04_3731_4C*	0.48	0.26	0.21	0.00
65	BMC_04_3731_4D*	-0.54	-0.19	0.26	-0.29
66	BMC_05_6782_1*	0.64	0.34	0.50	1.15
67	BMC_05_6782_9A*	0.49	0.65	0.53	1.09
68	BMC_05_6782_9B*	0.11	0.65	0.53	0.72
69	BMC_08_8598_11*	-1.00	0.46	0.68	1.69
70	BMC_08_8598_16*	0.74	0.72	0.72	0.33
71	BMC_08_8598_18*	0.46	0.67	0.72	1.15
72	BMC_09_2877_15*	0.52	0.88	0.70	0.34
73	BMC_09_2877_6*	1.52	0.94	0.90	0.20

*: external test set

Table S5. Dataset of 83 BCPs compounds with their cytotoxicity on KB3-1 cell line: experimental pIC_{50} value and predictive results

No	Name	pIC50/KB3	pIC50 /KB3-S34	pIC50 /KB3-E12	pIC50 /KB3-H34	pIC50 /KB3-EH32	pIC50 /KB3-61	pIC50 /KB3-2D
1	BMC_03_1475_11	-1.76	-0.25	-0.27	-0.73	-0.36	-0.43	-0.39
2	BMC_03_1475_12	0.41	-0.24	-0.83	-0.80	-0.79	-0.49	-0.02
3	BMC_03_1475_14	-1.00	-0.48	-0.20	-1.17	-0.44	-0.62	-0.35
4	BMC_03_1475_15	0.68	0.16	-0.29	0.23	-0.18	-0.15	-0.08
5	BMC_03_1475_2B	-1.08	-1.25	-1.57	-1.20	-1.42	0.34	0.29
6	BMC_03_1475_5	-0.81	-0.05	-0.39	-0.21	-0.38	-0.68	-1.17
7	BMC_03_1475_6	-1.43	-0.79	-1.27	-0.79	-1.15	-0.55	-0.34
8	BMC_03_1475_7	-0.51	0.18	-0.07	-0.34	-0.19	-0.57	-1.11
9	BMC_03_1475_8	-0.52	0.13	-0.09	-0.22	-0.16	-0.60	-1.39
10	BMC_03_1475_9	-1.45	-1.64	-1.49	-1.63	-1.43	-0.63	-0.87
11	BMC_03_3795_10A	0.80	0.07	0.83	0.58	0.60	0.34	0.32
12	BMC_03_3795_10C	-0.85	-0.35	0.06	-0.29	-0.17	-0.02	-0.09
13	BMC_03_3795_10D	0.74	0.79	0.78	0.77	0.61	0.31	0.38
14	BMC_03_3795_10E	-0.48	-0.19	0.22	0.15	0.07	0.13	0.71
15	BMC_03_3795_12B	0.64	-0.22	0.49	-0.06	0.33	0.32	-0.65
16	BMC_03_3795_12D	0.72	1.00	0.90	0.90	0.80	0.31	0.19
17	BMC_03_3795_3	-0.74	-1.35	-1.59	-0.88	-1.36	0.38	0.13
18	BMC_03_3795_5	1.30	0.24	0.17	-0.07	0.05	-0.34	0.39
19	BMC_04_5585_15B	2.52	1.84	1.71	2.13	2.06	2.12	1.76
20	BMC_04_5585_15D	2.22	2.22	2.03	2.32	2.07	2.47	1.96
21	BMC_05_6782_7A	1.22	0.93	1.13	0.81	0.88	1.02	1.48
22	BMC_05_6782_7B	1.47	1.40	1.13	1.16	1.05	1.10	1.13
23	BMC_05_6782_7C	1.26	1.87	1.05	1.20	0.96	1.21	1.25
24	BMC_05_6782_7D	1.35	1.12	1.20	1.43	1.18	1.33	1.35
25	BMC_05_6782_7E	0.33	0.53	0.95	0.74	0.87	1.14	0.93
26	BMC_05_6782_9C	2.22	1.69	1.55	1.71	1.61	1.04	1.57
27	BMC_05_6782_9D	0.35	0.55	0.92	0.84	0.91	0.98	1.08
28	BMC_05_6782_9E	1.52	1.46	1.47	1.41	1.50	1.08	1.70
29	BMC_05_6782_9F	1.22	0.95	1.04	1.22	1.01	0.61	0.59
30	BMC_05_6782_9G	0.30	0.40	1.15	0.42	1.04	0.18	0.55
31	BMC_05_6782_9H	2.52	1.61	1.49	1.55	1.58	1.21	1.70
32	BMC_05_6782_9I	1.74	1.57	1.50	1.50	1.57	1.25	2.05
33	BMC_05_6782_9J	1.00	1.45	1.49	1.19	1.56	1.42	1.72
34	BMC_05_6782_9K	1.35	1.90	1.25	1.32	1.33	1.05	1.26
35	BMC_05_6782_9L	0.60	0.23	0.88	0.61	0.89	1.23	1.33
36	BMC_06_3131_10B	2.22	2.13	1.98	2.03	1.92	2.28	1.77
37	BMC_06_3131_10C	2.52	2.22	2.05	2.18	1.99	2.31	2.20
38	BMC_06_3131_10F	2.40	2.23	2.03	2.28	2.08	2.58	1.76
39	BMC_06_3131_10G	1.35	2.10	1.93	2.18	2.08	2.25	1.96
40	BMC_06_3131_10I	1.52	1.97	2.02	2.00	1.97	2.45	2.04
41	BMC_06_3131_10J	1.40	1.55	1.15	1.73	1.42	1.53	1.50
42	BMC_06_3131_10K	1.42	1.36	2.00	1.70	1.90	1.86	0.90
43	BMC_06_3131_10L	2.40	1.45	1.99	1.97	2.11	2.10	1.75

No	Name	pIC50/KB3	pIC50 /KB3-S34	pIC50 /KB3-E12	pIC50 /KB3-H34	pIC50 /KB3-EH32	pIC50 /KB3-61	pIC50 /KB3-2D
44	BMC_06_3131_10M	2.22	1.84	2.00	1.92	2.02	2.17	2.02
45	BMC_08_8598_10	0.82	0.81	1.04	0.90	1.07	0.95	0.44
46	BMC_08_8598_12	1.52	1.02	1.28	1.52	1.47	1.02	1.10
47	BMC_08_8598_13	1.22	1.25	1.54	1.26	1.93	1.34	1.19
48	BMC_08_8598_14	0.74	0.90	0.98	0.79	0.85	0.90	0.99
49	BMC_08_8598_15	0.82	1.28	0.72	1.04	0.94	1.13	1.06
50	BMC_08_8598_17	1.68	1.69	1.47	1.59	1.92	1.28	1.21
51	BMC_08_8598_19	1.46	1.60	0.85	1.08	0.71	1.09	1.16
52	BMC_08_8598_21	0.64	1.04	1.34	0.99	1.14	0.94	0.88
53	BMC_08_8598_23	1.57	1.34	1.53	1.45	1.36	1.06	1.47
54	BMC_09_2877_10	0.52	1.23	1.02	1.04	1.03	1.04	1.70
55	BMC_09_2877_11	1.30	1.26	1.29	1.47	1.31	1.08	1.14
56	BMC_09_2877_12	1.60	1.83	1.16	1.57	1.21	1.08	1.05
57	BMC_09_2877_14	-0.18	0.79	1.14	1.06	1.17	0.85	1.24
58	BMC_09_2877_16	0.22	0.96	1.10	0.95	0.89	0.87	0.50
59	BMC_09_2877_17	0.80	0.92	0.89	1.21	0.85	0.82	1.25
60	BMC_09_2877_18	1.40	1.56	0.94	1.52	1.18	1.00	1.16
61	BMC_09_2877_19	0.49	0.37	0.89	0.42	0.50	0.80	1.01
62	BMC_09_2877_20	0.40	1.53	1.35	1.30	1.11	1.13	0.92
63	BMC_09_2877_21	0.77	0.52	0.96	0.90	1.15	1.34	1.13
64	BMC_09_2877_22	1.60	1.55	1.26	1.78	1.67	1.08	1.34
65	BMC_09_2877_3	2.52	2.00	2.03	2.08	2.00	2.25	1.65
66	BMC_09_2877_7	2.40	0.66	1.27	0.74	1.02	0.80	1.31
67	BMC_09_2877_8	1.52	1.29	1.04	1.12	1.21	0.99	0.88
68	BMC_09_2877_9*	1.52	0.97	1.15	1.17	1.05	0.81	1.64
69	BMC_03_3795_10B*	0.52	-0.36	-0.04	-0.29	-0.19	-0.06	-0.46
70	BMC_03_3795_10F*	0.12	-0.19	0.36	0.21	0.13	0.11	0.35
71	BMC_03_3795_12C*	-1.11	-0.22	-0.03	-0.15	-0.07	0.25	-0.28
72	BMC_03_3795_4*	0.52	0.28	0.94	0.63	0.76	0.40	0.13
73	BMC_05_6782_1*	2.30	0.99	0.97	0.92	1.06	2.23	1.01
74	BMC_05_6782_9A*	2.30	1.71	1.50	1.59	1.55	1.00	1.46
75	BMC_05_6782_9B*	2.52	1.82	1.38	1.60	1.51	1.02	1.58
76	BMC_06_3131_10D*	2.70	2.12	2.02	2.21	2.05	2.43	2.20
77	BMC_08_8598_11*	0.89	0.63	1.12	0.83	0.89	0.75	1.31
78	BMC_08_8598_16*	1.70	1.29	0.87	1.47	1.08	1.11	1.14
79	BMC_08_8598_18*	1.52	1.38	0.87	1.36	0.72	0.90	1.26
80	BMC_08_8598_20*	1.26	0.77	1.33	1.10	1.20	0.96	1.24
81	BMC_08_8598_22*	-0.08	1.16	1.35	1.10	1.13	0.94	0.51
82	BMC_09_2877_15*	1.51	0.98	1.15	1.02	1.05	0.87	0.88
83	BMC_09_2877_6*	1.35	0.87	1.05	0.77	0.95	0.99	0.44

*: external test set

Table S6. Dataset of 81 BCPs compounds with their cytotoxicity on KBV-1 cell line: experimental pIC_{50} value and predictive results

No	Name	pIC50/KBV	pIC50/KBV-S34	pIC50/KBV-71	pIC50/KBV-2D
1	BMC_03_1475_12	0.19	0.16	-0.20	0.53
2	BMC_03_1475_15	0.77	0.27	-0.04	1.48
3	BMC_03_1475_2B	-1.28	-1.01	0.18	-0.37
4	BMC_03_1475_5	-0.54	0.04	-0.32	-0.75
5	BMC_03_1475_6	-1.46	-0.91	-0.39	-1.21
6	BMC_03_1475_8	-0.40	0.11	-0.37	-0.85
7	BMC_03_1475_9	-1.88	-2.06	-1.02	-1.11
8	BMC_03_3795_10A	0.62	0.30	0.68	0.13
9	BMC_03_3795_10C	-0.34	0.21	0.49	0.20
10	BMC_03_3795_10D	0.62	0.66	0.05	-0.20
11	BMC_03_3795_10E	0.52	0.24	0.63	0.84
12	BMC_03_3795_12B	0.57	0.20	0.27	0.33
13	BMC_03_3795_12D	0.60	0.62	-0.43	-0.18
14	BMC_03_3795_3	-0.26	-1.03	0.20	0.45
15	BMC_03_3795_5	0.92	0.27	-0.22	0.52
16	BMC_04_5585_15B	2.15	1.09	1.52	0.93
17	BMC_05_6782_7A	0.51	0.48	0.58	0.81
18	BMC_05_6782_7B	0.66	0.49	0.55	0.55
19	BMC_05_6782_7C	1.00	0.87	0.58	0.37
20	BMC_05_6782_7D	0.64	0.54	0.66	0.32
21	BMC_05_6782_7E	-0.36	-0.17	0.58	-0.18
22	BMC_05_6782_9C	0.96	0.93	0.20	0.21
23	BMC_05_6782_9D	-0.26	0.07	0.15	-0.33
24	BMC_05_6782_9E	0.33	0.22	0.27	0.81
25	BMC_05_6782_9F	0.17	0.01	-0.42	0.20
26	BMC_05_6782_9G	-0.90	-0.92	-0.88	-0.24
27	BMC_05_6782_9H	0.82	0.21	0.21	0.41
28	BMC_05_6782_9I	0.74	0.01	0.33	0.42
29	BMC_05_6782_9J	-0.88	-0.22	-0.10	0.24
30	BMC_05_6782_9K	-0.65	0.58	0.26	-0.25
31	BMC_05_6782_9L	-0.30	-0.36	0.03	0.10
32	BMC_06_3131_10B	1.82	1.46	1.51	1.44
33	BMC_06_3131_10C	1.92	1.52	1.60	2.06
34	BMC_06_3131_10F	1.64	1.19	1.70	1.46
35	BMC_06_3131_10G	0.92	1.25	1.40	0.97
36	BMC_06_3131_10I	1.05	1.38	1.48	1.49
37	BMC_06_3131_10J	0.60	0.57	0.56	0.00
38	BMC_06_3131_10K	0.92	0.96	0.97	0.57
39	BMC_06_3131_10L	1.80	1.02	1.60	0.96
40	BMC_06_3131_10M	1.19	1.28	1.56	1.41
41	BMC_08_8598_10	0.40	0.49	0.40	0.68
42	BMC_08_8598_13	0.60	0.63	0.54	1.17
43	BMC_08_8598_14	0.35	0.46	0.05	-0.09

No	Name	pIC50/KBV	pIC50/KBV-S34	pIC50/KBV-71	pIC50/KBV-2D
44	BMC_08_8598_15	0.44	0.62	0.51	0.24
45	BMC_08_8598_19	0.40	0.68	0.51	0.39
46	BMC_08_8598_21	0.38	0.59	0.08	0.19
47	BMC_08_8598_23	-0.15	0.33	0.02	0.22
48	BMC_09_2877_10	0.36	0.86	0.52	0.60
49	BMC_09_2877_11	0.30	0.91	0.47	0.36
50	BMC_09_2877_12	1.10	1.29	0.48	0.48
51	BMC_09_2877_16	0.15	0.64	0.24	0.05
52	BMC_09_2877_17	0.43	0.33	0.28	0.90
53	BMC_09_2877_18	0.30	0.87	0.45	-0.24
54	BMC_09_2877_19	0.40	0.00	0.34	0.30
55	BMC_09_2877_20	-0.60	0.56	0.47	-0.23
56	BMC_09_2877_21	-0.28	-0.22	0.44	0.39
57	BMC_09_2877_22	1.57	0.57	0.39	1.54
58	BMC_09_2877_7	1.80	0.47	0.33	1.06
59	BMC_09_2877_8	0.77	0.49	0.54	1.04
60	BMC_09_2877_9	-0.25	0.49	0.18	0.04
61	BMC_03_1475_11*	-2.30	0.21	-0.15	0.46
62	BMC_03_1475_7*	-0.40	0.23	-0.32	-1.20
63	BMC_03_3795_10B*	0.40	0.21	0.53	0.21
64	BMC_03_3795_10F*	0.44	0.24	0.63	0.93
65	BMC_03_3795_12C*	-0.36	0.15	0.22	0.36
66	BMC_03_3795_4*	0.70	0.27	0.20	0.46
67	BMC_04_5585_15D*	2.22	1.28	1.65	0.76
68	BMC_05_6782_1*	2.30	0.31	1.18	0.90
69	BMC_05_6782_9A*	0.66	0.64	0.21	0.36
70	BMC_05_6782_9B*	0.49	0.81	0.21	0.64
71	BMC_06_3131_10D*	2.40	1.30	1.54	1.49
72	BMC_08_8598_11*	0.52	0.37	0.20	0.67
73	BMC_08_8598_12*	0.80	0.55	0.44	0.22
74	BMC_08_8598_16*	0.30	0.64	0.43	-0.07
75	BMC_08_8598_17*	1.22	0.73	0.57	0.90
76	BMC_08_8598_18*	0.48	0.47	0.13	0.78
77	BMC_08_8598_20*	0.40	0.50	0.06	0.14
78	BMC_08_8598_22*	-0.60	0.64	0.04	-0.30
79	BMC_09_2877_15*	1.00	0.61	0.27	0.23
80	BMC_09_2877_3*	2.40	1.35	1.54	0.85
81	BMC_09_2877_6*	1.12	0.56	0.39	0.93

*: external test set

Table S7. Dataset of 60 BCPs compounds with their cytotoxicity on KBH5.0 cell line: experimental pIC_{50} value and predictive results

No	Name	pIC50/KBH	pIC50 /KBH-CoMFAS43	pIC50 /KBH-S43	pIC50 /KBH-59	pIC50 /KBH-2D
1	BMC_04_5585_15B	2.40	1.60	1.45	1.91	1.65
2	BMC_04_5585_15D	2.15	1.79	1.68	1.86	1.69
3	BMC_05_6782_7A	0.92	0.94	0.82	0.98	1.03
4	BMC_05_6782_7B	1.14	1.09	0.95	0.97	0.78
5	BMC_05_6782_7C	1.19	1.13	1.26	0.93	0.75
6	BMC_05_6782_7D	0.96	0.94	0.97	0.83	0.72
7	BMC_05_6782_7E	0.28	0.40	0.26	0.93	0.83
8	BMC_05_6782_9C	1.30	1.39	1.32	0.78	1.44
9	BMC_05_6782_9D	0.05	0.12	0.43	0.60	0.69
10	BMC_05_6782_9E	1.15	0.76	0.68	0.80	1.13
11	BMC_05_6782_9F	0.46	0.46	0.51	-0.11	-0.10
12	BMC_05_6782_9G	-0.81	-0.98	-0.61	-0.74	-0.55
13	BMC_05_6782_9H	1.30	0.93	0.68	0.86	1.16
14	BMC_05_6782_9I	1.40	0.82	0.51	0.88	1.21
15	BMC_05_6782_9J	-0.18	0.29	0.32	0.29	0.10
16	BMC_05_6782_9K	0.30	0.92	1.17	0.82	1.34
17	BMC_05_6782_9L	0.19	-0.01	0.11	0.38	0.85
18	BMC_06_3131_10B	2.05	1.87	1.73	1.70	1.76
19	BMC_06_3131_10C	2.30	2.13	1.87	1.79	1.61
20	BMC_06_3131_10F	1.66	1.66	1.60	1.84	1.66
21	BMC_06_3131_10G	0.82	1.59	1.62	1.63	0.93
22	BMC_06_3131_10I	1.30	1.44	1.63	1.67	1.84
23	BMC_06_3131_10J	0.77	1.00	0.84	0.70	0.58
24	BMC_06_3131_10K	1.15	1.23	1.22	1.07	0.72
25	BMC_06_3131_10L	1.70	1.19	1.26	1.96	1.68
26	BMC_06_3131_10M	1.07	1.70	1.56	1.88	1.57
27	BMC_08_8598_10	0.40	0.53	0.54	0.55	0.45
28	BMC_08_8598_12	0.68	0.62	0.57	0.62	0.96
29	BMC_08_8598_13	0.54	0.57	0.57	0.53	0.24
30	BMC_08_8598_14	0.38	0.48	0.49	0.84	0.83
31	BMC_08_8598_15	0.49	0.58	0.58	0.59	0.46
32	BMC_08_8598_19	0.89	0.95	0.90	0.60	0.53
33	BMC_08_8598_21	0.32	0.64	0.63	0.69	1.00
34	BMC_08_8598_23	0.70	0.68	0.58	0.58	0.99
35	BMC_09_2877_10	0.31	0.91	1.14	0.69	0.46
36	BMC_09_2877_11	0.70	0.99	1.19	0.72	0.93
37	BMC_09_2877_12	1.15	1.63	1.64	0.77	0.51
38	BMC_09_2877_16	0.42	0.58	0.92	0.80	0.53
39	BMC_09_2877_17	0.54	0.67	0.77	1.01	1.07
40	BMC_09_2877_18	0.74	1.31	1.25	0.63	0.81
41	BMC_09_2877_19	0.49	0.30	0.26	1.02	0.98
42	BMC_09_2877_20	0.02	0.80	0.95	0.67	0.86
43	BMC_09_2877_21	0.26	0.05	0.30	0.46	0.52

No	Name	pIC50/KBH	pIC50 /KBH-CoMFAS43	pIC50 /KBH-S43	pIC50 /KBH-59	pIC50 /KBH-2D
44	BMC_09_2877_22	1.42	1.06	0.99	0.65	0.40
45	BMC_09_2877_3	2.40	1.88	1.68	1.79	1.52
46	BMC_09_2877_7	1.72	0.88	0.73	1.12	1.32
47	BMC_09_2877_8	1.22	0.88	0.87	0.66	0.89
48	BMC_09_2877_9	1.40	0.88	0.89	1.04	0.87
49	BMC_05_6782_1*	2.22	0.50	0.44	1.43	1.47
50	BMC_05_6782_9A*	1.22	1.22	1.10	0.78	1.03
51	BMC_05_6782_9B*	1.70	1.28	1.27	0.77	1.36
52	BMC_06_3131_10D*	2.40	1.76	1.66	1.85	1.64
53	BMC_08_8598_11*	0.54	0.78	0.58	0.94	1.27
54	BMC_08_8598_16*	0.96	0.66	0.61	0.64	0.93
55	BMC_08_8598_17*	1.40	0.67	0.63	0.66	-0.05
56	BMC_08_8598_18*	1.15	0.52	0.64	0.82	1.02
57	BMC_08_8598_20*	-0.43	0.55	0.55	0.77	1.55
58	BMC_08_8598_22*	-0.56	0.56	0.59	0.60	0.49
59	BMC_09_2877_15*	1.10	0.82	0.90	0.89	1.04
60	BMC_09_2877_6*	1.10	0.88	0.82	0.81	0.45

*: External test set

Table S8. Dataset of 94 BCPs compounds with experimental topoisomerase-1 inhibitory value and predictive results

No	Name	pIC50/TOP1	pIC50/TOP1-E34	pIC50/TOP1-H64	pIC50/TOP1-307	pIC50/TOP1-2D
1	BMC_03_1475_12	-1.78	-2.11	-2.31	-1.68	-1.38
2	BMC_03_1475_14	-2.30	-2.32	-2.61	-1.71	-2.09
3	BMC_03_1475_15	-1.30	-1.26	-1.12	-1.71	-0.74
4	BMC_03_1475_2B	-1.30	-2.15	-1.92	-0.62	-1.63
5	BMC_03_1475_5	-1.30	-1.30	-1.64	-2.01	-2.14
6	BMC_03_1475_8	-2.00	-1.03	-1.43	-1.76	-0.94
7	BMC_03_1809_6	-2.20	-1.99	-1.75	-1.48	-1.91
8	BMC_03_3795_10A	-0.90	-1.45	-1.25	-1.58	-1.25
9	BMC_03_3795_10C	-2.30	-2.30	-2.36	-2.08	-1.82
10	BMC_03_3795_10E	-2.70	-1.98	-1.88	-1.78	-1.68
11	BMC_03_3795_3	-2.00	-2.36	-1.98	-0.79	-1.42
12	BMC_03_3795_5	-0.90	-0.99	-1.35	-1.20	-0.82
13	BMC_03_521_7C	-2.20	-1.17	-1.11	-1.66	-1.66
14	BMC_03_521_7G	-0.60	-1.22	-1.04	-1.39	-1.82
15	BMC_04_3731_2	0.52	0.05	0.20	-0.58	-0.57
16	BMC_04_3731_3A	-0.95	-0.89	-1.03	-0.79	-1.59
17	BMC_04_3731_3B	-0.78	-0.71	-1.00	-1.02	-0.83
18	BMC_04_3731_4A	-2.00	-1.11	-1.42	-1.19	-1.47
19	BMC_04_3731_4B	-1.08	-0.60	-0.82	-1.15	-0.71
20	BMC_04_3731_5	-1.00	-0.82	-1.09	-1.26	-1.23
21	BMC_04_5585_15B	0.22	0.31	0.36	0.36	0.38
22	BMC_04_5585_15D	1.70	0.29	-0.11	0.55	-0.21
23	BMC_04_795_1B	0.52	0.05	0.21	-0.44	0.12
24	BMC_04_795_1C	-2.00	-1.92	-1.07	-0.65	-0.48
25	BMC_04_795_1D	0.30	-0.02	-0.29	-0.37	0.24
26	BMC_04_795_1F	0.70	-0.12	-0.03	-0.34	-0.20
27	BMC_04_795_1G	-0.30	-0.18	-0.08	-0.29	-0.38
28	BMC_04_795_1H	-1.30	-0.44	-0.73	-0.71	-0.94
29	BMC_04_795_2	-1.00	-1.27	-1.15	-1.39	-0.80
30	BMC_05_6782_7A	-1.00	-0.52	-0.81	-0.63	-0.72
31	BMC_05_6782_7B	-0.70	-0.12	-0.47	-0.70	-0.37
32	BMC_05_6782_7C	0.00	0.21	-0.46	-0.77	-0.67
33	BMC_05_6782_7D	-0.90	-1.18	-0.45	-0.94	-0.54
34	BMC_05_6782_7E	-0.81	-0.98	-0.80	-0.66	-0.58
35	BMC_05_6782_9C	0.40	-0.18	-0.06	-0.09	0.15
36	BMC_05_6782_9D	-1.00	-0.94	-0.44	-0.18	-0.50
37	BMC_05_6782_9E	0.15	-0.43	-0.48	-0.06	0.66
38	BMC_05_6782_9F	-1.08	-1.06	-0.17	-1.30	-1.48
39	BMC_05_6782_9G	-1.78	-1.85	-1.54	-2.36	-1.79
40	BMC_05_6782_9H	0.22	-0.05	-0.11	0.04	0.58
41	BMC_05_6782_9I	-0.18	-0.15	-0.23	0.09	0.28
42	BMC_05_6782_9J	0.52	-0.09	0.37	-0.33	-0.21

No	Name	pIC50/TOP1	pIC50/TOP1-E34	pIC50/TOP1-H64	pIC50/TOP1-307	pIC50/TOP1-2D
43	BMC_05_6782_9K	-0.70	0.05	-0.44	-0.15	0.34
44	BMC_05_6782_9L	-0.48	-0.86	-0.50	-0.29	-0.61
45	BMC_06_3131_10B	-0.08	0.49	0.33	0.38	0.21
46	BMC_06_3131_10C	0.70	0.39	0.17	0.40	0.73
47	BMC_06_3131_10F	-0.48	0.14	-0.19	0.60	-0.37
48	BMC_06_3131_10G	-0.95	0.28	0.28	0.23	-0.38
49	BMC_06_3131_10I	0.35	0.32	0.33	0.60	0.00
50	BMC_06_3131_10K	-1.11	-0.60	-0.63	-0.86	-1.01
51	BMC_06_3131_10L	0.46	0.18	0.53	0.32	0.36
52	BMC_06_3131_10M	0.82	0.37	0.40	0.30	0.50
53	BMC_08_8598_10	0.70	-0.18	-0.01	0.08	-0.67
54	BMC_08_8598_12	0.70	0.09	1.06	0.10	0.33
55	BMC_08_8598_13	0.70	0.40	0.60	0.67	0.30
56	BMC_08_8598_17	0.40	0.69	0.85	0.77	-0.15
57	BMC_08_8598_19	0.10	0.45	-0.22	0.19	-0.30
58	BMC_08_8598_21	0.10	0.12	0.44	0.40	-0.03
59	BMC_08_8598_23	0.70	0.10	0.98	0.35	0.63
60	BMC_09_2877_10	-0.89	-0.05	-0.54	-0.21	-0.48
61	BMC_09_2877_11	0.52	0.02	-0.03	-0.27	0.24
62	BMC_09_2877_12	0.30	0.25	-0.16	-0.24	-0.24
63	BMC_09_2877_14	0.10	-0.38	-0.31	-0.24	0.13
64	BMC_09_2877_16	-0.04	-0.34	-0.45	-0.09	-0.55
65	BMC_09_2877_17	-0.89	-0.73	-0.97	-0.79	-0.52
66	BMC_09_2877_18	-1.00	0.01	-0.31	-0.32	-0.50
67	BMC_09_2877_19	-1.18	-1.13	-1.09	-0.79	-0.64
68	BMC_09_2877_20	0.70	0.14	-0.40	-0.21	-0.29
69	BMC_09_2877_21	-0.96	-0.89	-0.42	-0.18	-0.13
70	BMC_09_2877_22	-0.28	0.08	0.06	-0.40	0.65
71	BMC_09_2877_3	0.22	0.39	0.41	0.26	0.07
72	BMC_09_2877_7	-0.67	-0.52	-0.55	-0.58	-0.60
73	BMC_09_2877_8	-0.04	-0.08	-0.73	-0.21	-0.35
74	BMC_09_2877_9	-0.96	-0.38	-0.62	-0.78	-1.07
75	BMC_03_1475_11*	-3.00	-1.81	-2.12	-1.74	-1.15
76	BMC_03_1475_7*	-1.30	-0.99	-1.44	-1.80	-0.75
77	BMC_03_1809_7*	-2.30	-2.31	-1.99	-1.36	-1.91
78	BMC_03_3795_10B*	-2.30	-2.07	-2.35	-2.04	-1.83
79	BMC_03_3795_10F*	-1.00	-1.73	-1.80	-1.73	-1.66
80	BMC_03_3795_12C*	-2.00	-2.07	-1.85	-1.25	-2.00
81	BMC_03_3795_4*	-1.00	-0.95	-0.82	-0.78	-1.44
82	BMC_03_521_7F*	-1.30	-1.20	-0.94	-1.36	-1.60
83	BMC_04_3731_3C*	-0.30	-0.88	-1.10	-1.06	-0.98
84	BMC_04_3731_4C*	-0.78	-0.87	-1.12	-1.11	-0.86
85	BMC_04_795_1E*	-0.70	-0.98	-1.22	-0.18	-0.93
86	BMC_05_6782_1*	0.52	-0.51	-0.79	-0.60	0.01
87	BMC_05_6782_9A*	0.30	-0.03	-0.15	-0.18	0.49
88	BMC_05_6782_9B*	0.22	-0.15	-0.31	-0.22	0.15

No	Name	pIC50/TOP1	pIC50/TOP1-E34	pIC50/TOP1-H64	pIC50/TOP1-307	pIC50/TOP1-2D
89	BMC_06_3131_10D*	1.15	0.32	0.03	0.49	0.55
90	BMC_08_8598_11*	0.10	-0.54	-0.23	-0.19	-0.51
91	BMC_08_8598_16*	0.00	0.52	0.94	0.05	0.08
92	BMC_08_8598_20*	-0.04	-0.23	0.68	0.25	0.22
93	BMC_09_2877_15*	-0.08	-0.31	-0.44	-0.15	-0.12
94	BMC_09_2877_6*	0.60	-0.34	-1.01	-0.31	-0.77

***: External test set**

Model 1. 2D QSAR model on RPMI8402 cytotoxicity

No	Desciptor	1	2	3	4	5	6	7	Y=		p
1	Petitjean	1,00							-8,714	-0,232	0,001
2	BCUT_PEOE_0	0,18	1,00						-2,807	-0,389	0,000
3	GCUT_PEOE_1	-0,18	-0,61	1,00					-7,865	-0,146	0,039
4	a_ICM	0,10	-0,31	-0,04	1,00				14,106	0,766	0,000
5	a_nN	-0,03	-0,71	0,39	0,64	1,00			-0,264	-0,223	0,001
6	PEOE_VSA+3	0,06	-0,32	0,15	0,25	0,09	1,00		0,043	0,348	0,000
7	Density	0,17	0,15	-0,40	0,78	0,09	0,31	1,00	-20,714	-0,414	0,000
	Coff								-11,878		

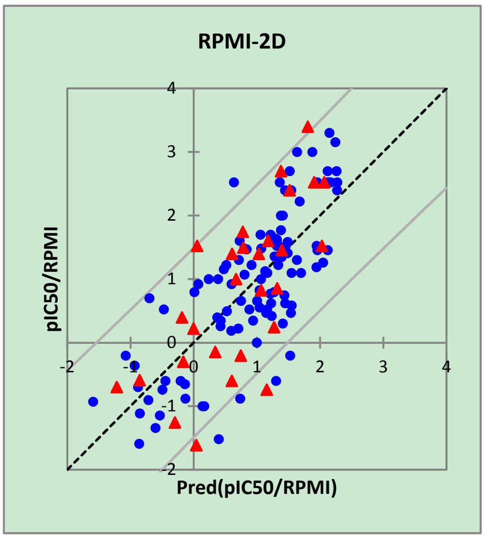

The relationship between observed and predicted data from QSAR model and its 95% confidence interval of RPMI8402 cell line

Model 2. 2D QSAR model on CPTk5-2D

No	Descriptor	1	2	3	4	5	6	7	8	9	Y=		p
1	GCUT_SMR_0	1,00									6,678	0,232	0,018
2	a_aro	-0,11	1,00								-0,342	-0,882	0,000
3	chi1v_C	-0,33	0,66	1,00							-0,421	-0,445	0,000
4	a_nN	-0,20	-0,33	0,04	1,00						0,503	0,588	0,000
5	PEOE_VSA+1	-0,66	0,53	0,39	-0,08	1,00					0,029	0,765	0,000
6	PEOE_VSA+2	-0,40	0,13	0,25	0,25	0,18	1,00				0,037	0,369	0,000
7	PEOE_VSA+3	-0,35	-0,07	0,25	0,16	-0,15	0,47	1,00			0,075	0,841	0,000
8	a_acc	-0,14	-0,24	0,23	0,72	-0,09	0,48	0,51	1,00		-0,326	-0,494	0,000
9	SlogP	0,15	0,78	0,64	-0,57	0,21	-0,10	-0,15	-0,36	1,00	1,027	0,995	0,000
	Coff										3,593		

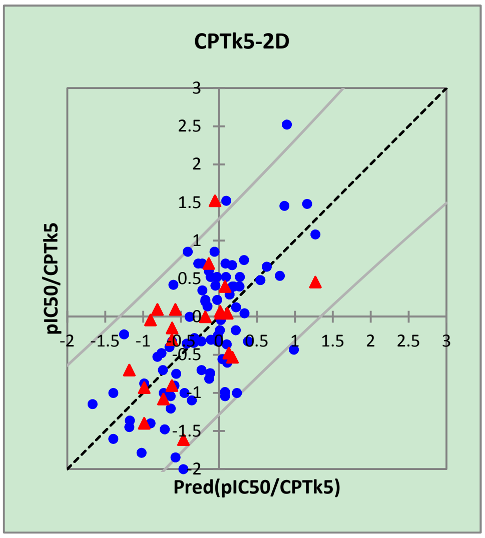

The relationship between observed and predicted data from QSAR model and its 95% confidence interval of CPTk5 cell line

Model 3. 2D QSAR on P388

No	Descriptor	1	2	3	4	5	6	7	8	9	10	Y=		p
1	diameter	1,00										0,566	0,808	0,000
2	petitjean	0,10	1,00									-7,136	-0,251	0,014
3	GCUT_SLOGP_0	0,05	0,04	1,00								1,051	0,338	0,001
4	a_nO	0,28	-0,05	0,30	1,00							-2,683	-26,447	0,000
5	PEOE_VSA+3	0,30	0,14	-0,11	0,19	1,00						-0,147	-14,915	0,000
6	PEOE_VSA-0	0,23	-0,22	0,13	0,11	0,08	1,00					0,022	6,890	0,000
7	PEOE_VSA_NEG	0,51	-0,21	0,29	0,17	-0,12	0,64	1,00				0,112	37,212	0,000
5	vsa_acc	0,15	0,07	0,25	0,39	0,02	0,03	0,28	1,00			0,419	45,254	0,000
9	vsa_other	0,15	-0,02	0,33	0,84	0,17	-0,07	0,12	0,32	1,00		0,147	18,039	0,000
10	SlogP	0,58	-0,11	0,21	0,16	0,26	0,18	0,44	-0,34	0,06	1,00	-1,681	-15,893	0,000
	coff											-15,908		

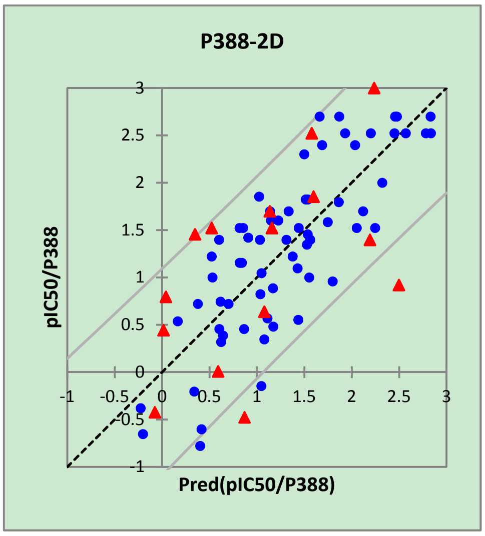

The relationship between observed and predicted data from QSAR model and its 95% confidence interval of P388 cell line

Model 4. 2D QSAR on CPT45-2D

No	descriptor	1	2	3	4	5	6	7	8	9	Y=		p
1	BCUT_PEOE_1	1,00									-19,423	-0,973	0,000
2	GCUT_PEOE_1	0,75	1,00								20,756	0,514	0,000
3	chi1v_C	-0,22	0,03	1,00							-1,699	-1,988	0,000
4	PEOE_VSA_FHYD	0,03	0,16	0,25	1,00						19,864	0,957	0,000
5	PEOE_VSA_FNEG	-0,03	0,05	0,13	0,00	1,00					-24,092	-1,376	0,000
6	PEOE_VSA_NEG	0,28	0,33	0,56	-0,03	0,64	1,00				0,065	2,310	0,000
7	vsa_other	-0,04	-0,28	0,11	-0,66	-0,01	0,15	1,00			0,056	0,735	0,000
8	SlogP_VSA1	0,07	-0,15	-0,05	-0,33	0,14	0,33	0,41	1,00		-0,060	-0,673	0,000
9	SlogP_VSA5	0,71	0,68	-0,17	0,08	-0,08	0,29	-0,20	-0,09	1,00	-0,013	-0,629	0,000
	Coff										-8,798		

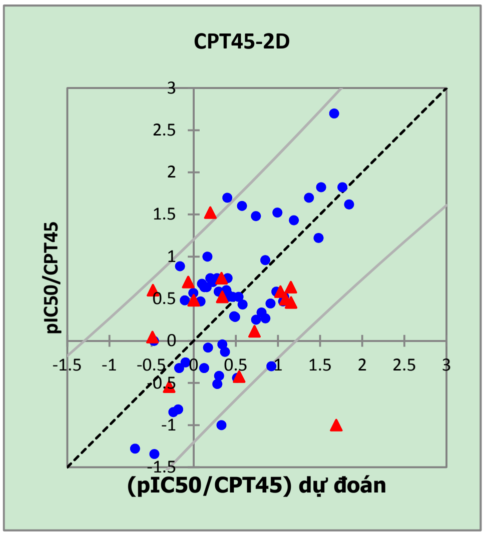

The relationship between observed and predicted data from QSAR model and its 95% confidence interval of CPT45 cell line

Model 5. 2D QSAR on KB3-2D

No	Descriptor	1	2	3	4	5	6	Y=		P
1	petitjean	1,00						-7,740	-0,227	0,008
2	VDistMa	-0,21	1,00					1,234	0,544	0,000
3	a_aro	0,13	0,41	1,00				-0,190	-0,452	0,000
4	PEOE_VSA+2	0,05	0,40	0,22	1,00			0,038	0,261	0,002
5	PEOE_VSA+3	0,16	0,32	-0,03	0,14	1,00		0,039	0,283	0,001
6	SlogP_VSA5	-0,30	0,42	-0,11	0,18	-0,01	1,00	-0,005	-0,190	0,026
	Coff							-5,430		

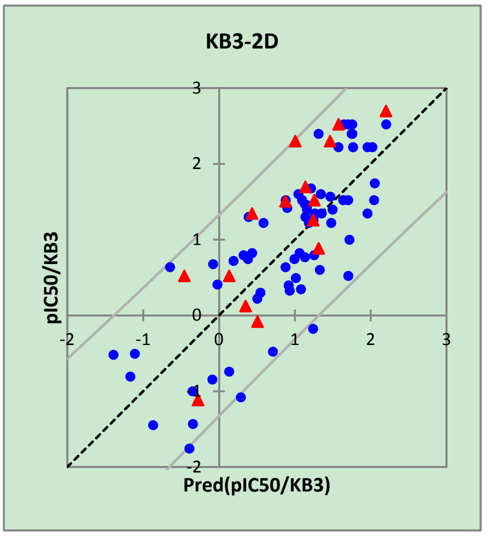

The relationship between observed and predicted data from QSAR model and its 95% confidence interval of KB3 cell line

Model 6. 2D QSAR on KBV cell line

No	Descriptor	1	2	3	4	5	6	7	8	Y=		P
1	BCUT_PEOE_2	1,00								9,713	0,761	0,000
2	GCUT_PEOE_1	-0,38	1,00							12,877	0,308	0,002
3	a_aro	0,42	0,25	1,00						-0,193	-0,621	0,000
4	PEOE_VSA+3	-0,23	0,17	-0,03	1,00					0,035	0,308	0,002
5	opr_leadlike	0,12	-0,28	-0,41	-0,12	1,00				0,728	0,441	0,000
6	a_acc	-0,50	0,18	-0,01	0,28	-0,39	1,00			0,597	0,741	0,000
7	vsa_pol	-0,08	-0,24	0,02	0,14	-0,09	0,72	1,00		-0,033	-0,328	0,001
8	SlogP_VSA9	-0,14	0,12	0,27	0,13	-0,28	0,28	0,19	1,00	0,017	0,392	0,000
	Coff									0,092		

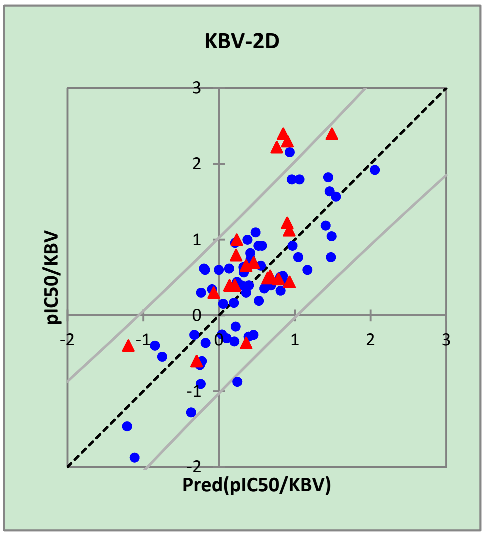

The relationship between observed and predicted data from QSAR model and its 95% confidence interval of KBV cell line

Model 7. 2D QSAR model on KBH

No	Descriptor	1	2	3	4	5	6	Y=	Chuẩn	p
1	a_aro	1,00						-0,271	-1,040	0,000
2	a_nN	0,07	1,00					0,665	0,656	0,000
3	a_nO	-0,02	-0,02	1,00				-0,278	-0,268	0,026
4	PEOE_VSA+0	0,02	0,77	0,03	1,00			-0,019	-0,935	0,000
5	vsa_acc	0,06	0,52	0,46	0,52	1,00		0,065	0,635	0,000
6	SlogP	0,76	-0,12	0,03	0,11	-0,07	1,00	0,546	0,632	0,000
	Coff							4,195		

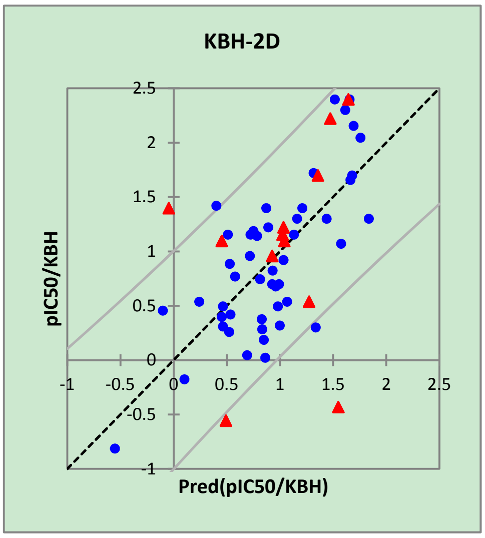

The relationship between observed and predicted data from QSAR model and its 95% confidence interval of KBH cell line

Model 8. 2D QSAR on Topoisomerase 1 inhibitory activity

No	Descriptor	1	2	3	4	5	6	7	8	9	10	Y=		p
1	GCUT_SLOGP_1	1,00										-13,772	-0,306	0,001
2	a_ICM	-0,44	1,00									27,553	1,597	0,000
3	b_1rotR	0,50	0,06	1,00								7,250	0,351	0,000
4	a_nN	0,04	0,65	0,51	1,00							-0,567	-0,577	0,000
5	PEOE_VSA+3	-0,33	0,04	0,06	-0,08	1,00						0,038	0,368	0,000
6	PEOE_VSA-1	0,32	-0,26	-0,01	0,02	-0,22	1,00					-0,021	-0,477	0,000
7	opr_leadlike	-0,16	-0,04	-0,45	-0,35	-0,26	-0,04	1,00				0,459	0,237	0,008
8	SlogP	0,52	-0,62	0,25	-0,40	0,11	0,47	-0,32	1,00			0,393	0,320	0,000
9	SlogP_VSA5	0,54	-0,14	0,66	0,21	-0,06	-0,33	-0,13	0,04	1,00		-0,013	-0,501	0,000
10	density	-0,67	0,74	-0,45	0,03	0,19	-0,25	0,15	-0,42	-0,54	1,00	-71,331	-1,546	0,000
	Coff											5,063		

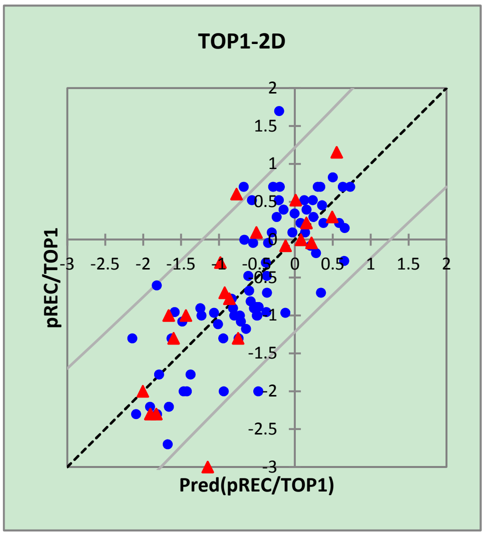

The relationship between observed and predicted data from QSAR model and its 95% confidence interval of topoisomerase -1 inhibitory activity

Model 9. Hologram QSAR on RPMI-151

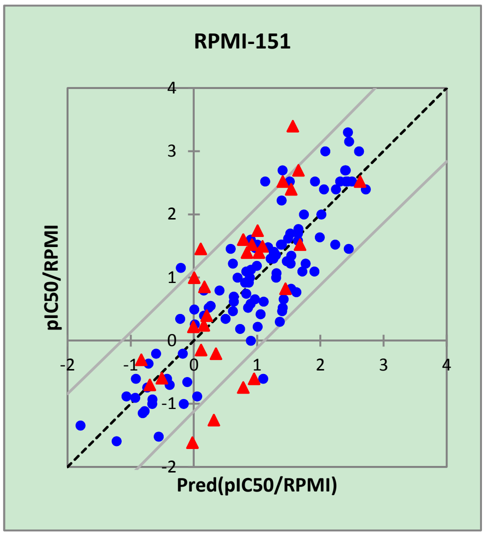

The relationship between observed and predicted data from QSAR model and its 95% confidence interval of RPMI cell line

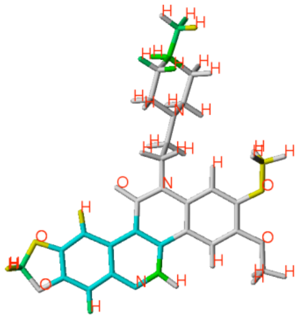

BMC_06_3131_10F
Exp: 2,39790
Pred: 2,71781

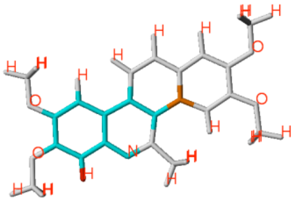

BMC_03_1809_5B

Exp: -1,34240
Pred: -1,79618

Model 10. Hologram QSAR on CPTk5-53

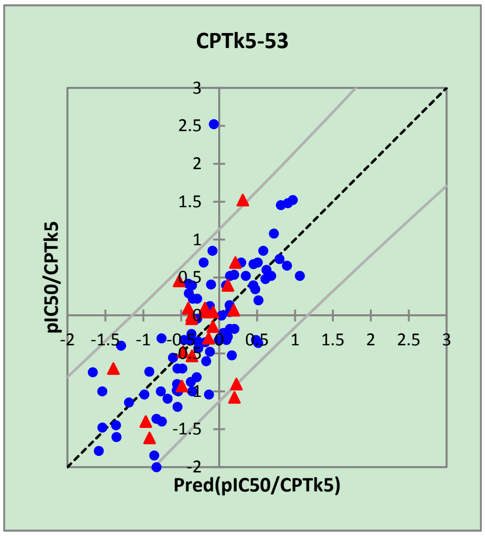

The relationship between observed and predicted data from QSAR model and its 95% confidence interval of CPTk5 cell line

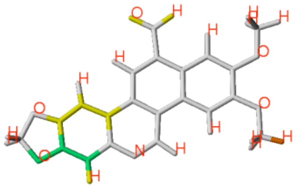

BMC_09_2877_7
Exp 2,52290
Pred: -0,07129

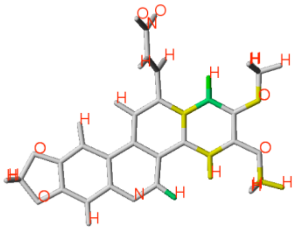

BMC_03_1475_12
Exp: -2,00
Pred: -0,82801

Model 11. Hologram QSAR on P388-353

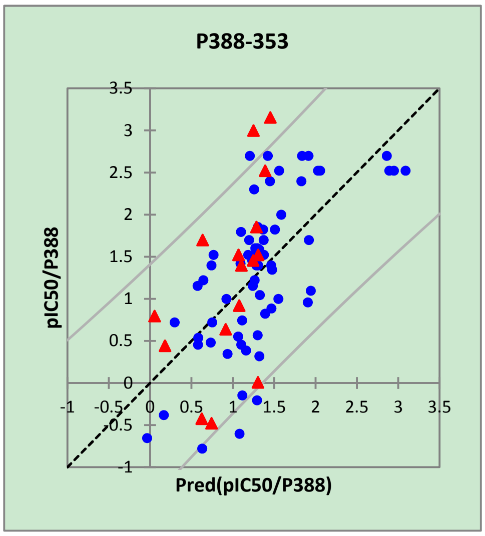

The relationship between observed and predicted data from QSAR model and its 95% confidence interval of P338 cell line

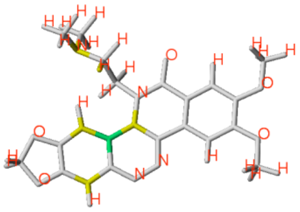

BMC_04_3731_2

Exp: 2,69900
Pred: 2,16123

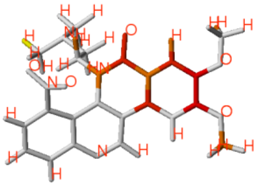

BMC_04_3731_3D
Exp: -0,77820
Pred: -1,04515

Model 12. Hologram QSAR on CPT45-199

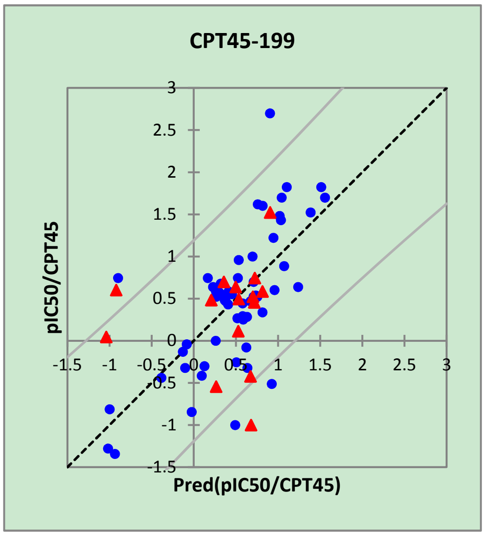

The relationship between observed and predicted data from QSAR model and its 95% confidence interval of CPT45 cell line

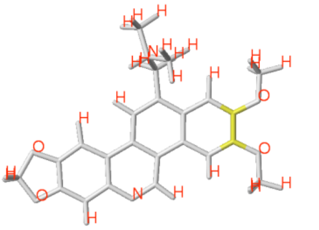

BMC_09_2877_8
Exp: 1,82390
Pred: 1,10111

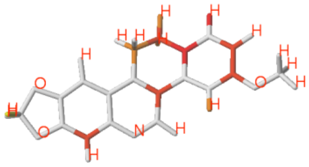

BMC_03_3795_10C
Exp: -1,34240
Pred: -0,93813

Model 13. Hologram QSAR on KB3-61

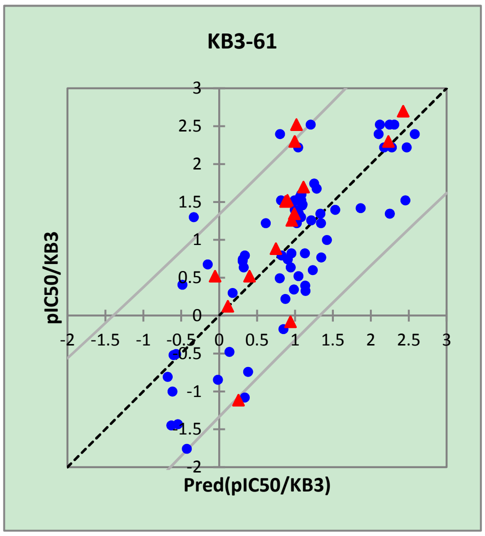

The relationship between observed and predicted data from QSAR model and its 95% confidence interval of KB3 cell line

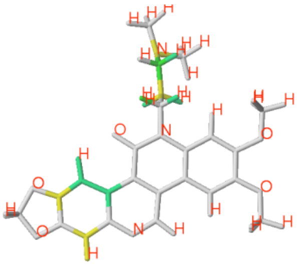

BMC_04_5585_15B
Exp: 2,52290
Pred: 2,11754

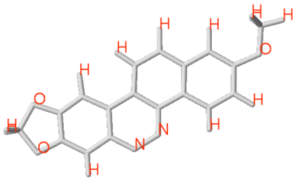

BMC_03_1475_11
Exp: -1,75590
Pred: -0,42833

Model 14. Hologram QSAR on KBV-71

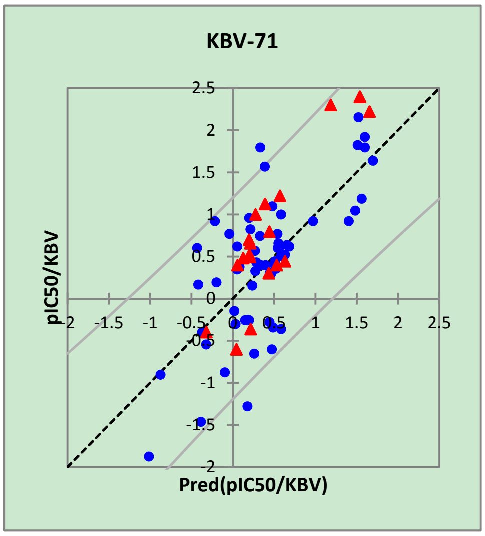

The relationship between observed and predicted data from QSAR model and its 95% confidence interval of KBV cell line

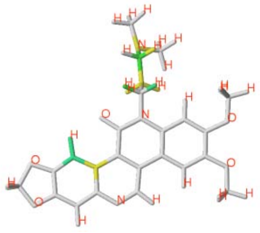

BMC_04_5585_15B
Exp: 2,15490
Pred:1,52010

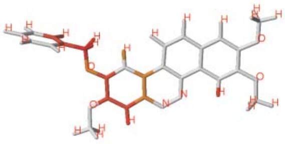

BMC_03_1475_11
Exp: -1,87510
Pred: -1,01580

Model 15. Hologram QSAR on KBH-59

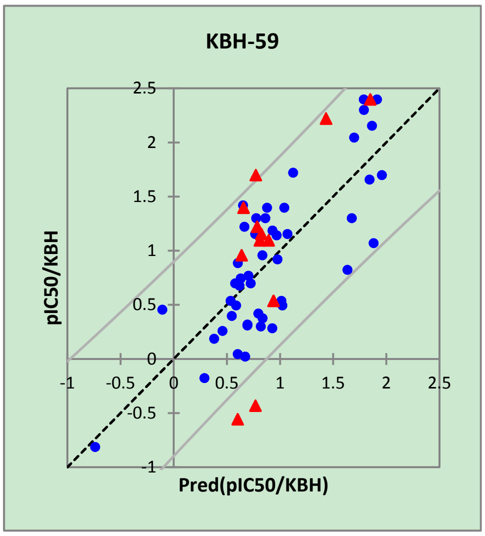

The relationship between observed and predicted data from QSAR model and its 95% confidence interval of KBH cell line

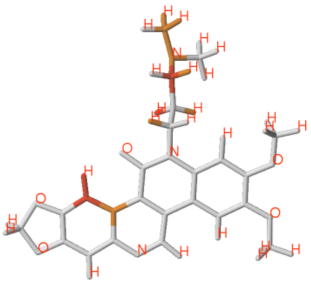

BMC_04_5585_15B
Exp: 2,39790
Pred:1,91122

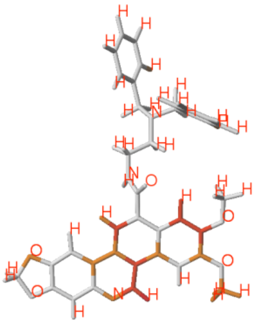

BMC_05_6782_9G
Exp: -0,81290
Pred: -0,73965

Model 16. Hologram QSAR on TOP1 inhibitory activity

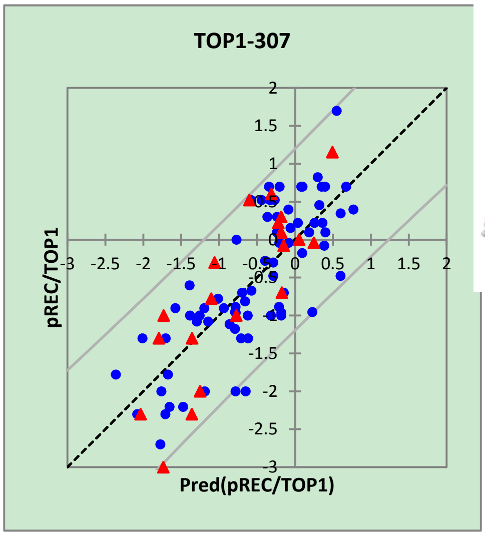

The relationship between observed and predicted data from QSAR model and its 95% confidence interval of Top1 inhibitory activity

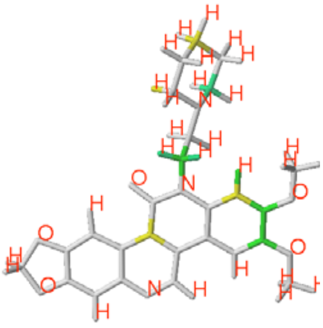

BMC_04_5585_15D

Exp: 1,69900
Pred:0,5483835

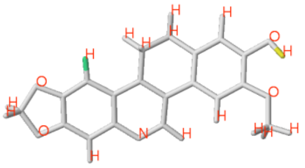

BMC_03_3795_10E

Exp: -2,69900
Pred: -1,77674

Model 17-18. 3D-QSAR on RPMI cytotoxicity

RPMI	q^2 for each column filter values				
**Descriptor fiels**	1	2	3	4	5
**S**	0,573	0,572	0,568	0,57	0,562
**E**	0,491	0,491	0,49	0,474	0,469
**H**	0,494	0,493	0,488	0,477	0,461
**D**	0	0	0	0	0
**A**	0,279	0,279	0,278	0,279	0,275
**s,e**	0,555	0,557	0,559	0,551	0,55
**s,h**	0,546	0,55	0,549	0,545	0,542
**s,d**	0,554	0,552	0,524	0,536	0,56
**s,a**	0,496	0,499	0,502	0,493	0,487
**e,h**	0,526	0,526	0,525	0,517	0,508
**e,d**	0,458	0,456	0,447	0,443	0,457
**e,a**	0,478	0,477	0,468	0,456	0,452
**h,d**	0,454	0,444	0,436	0,446	0,456
**h,a**	0,425	0,423	0,404	0,4	0,392
**d,a**	0,242	0,246	0,258	0,253	0,267
**s,e,h**	0,543	0,545	0,546	0,539	0,539
**s,e,d**	0,531	0,534	0,528	0,522	0,534
**s,e,a**	0,524	0,525	0,515	0,507	0,511
**s,h,d**	0,529	0,532	0,52	0,524	0,545
**s,h,a**	0,473	0,477	0,473	0,47	0,471
**s,d,a**	0,451	0,455	0,44	0,447	0,498
**e,h,d**	0,493	0,492	0,484	0,47	0,484

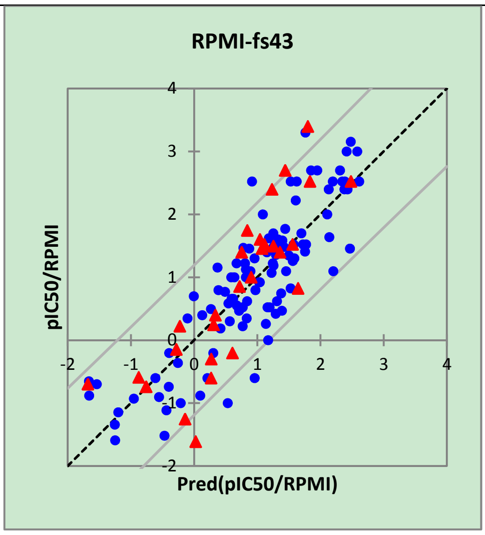

**e,h,a**	0,495	0,494	0,489	0,481	0,477
**e,d,a**	0,444	0,448	0,435	0,416	0,467
**h,d,a**	0,375	0,375	0,373	0,363	0,385
**s,e,h,d**	0,526	0,526	0,52	0,511	0,522
**s,e,h,a**	0,519	0,519	0,51	0,507	0,507
**s,e,d,a**	0,494	0,496	0,485	0,474	0,501
**s,h,d,a**	0,469	0,469	0,451	0,445	0,466
**e,h,d,a**	0,467	0,466	0,452	0,434	0,464
**s,e,h,d,a**	0,495	0,498	0,491	0,484	0,495
**CoMFA**	0,579	0,581	0,585	0,581	0,574
**CoMFA(s)**	0,609	0,609	0,608	0,607	0,597

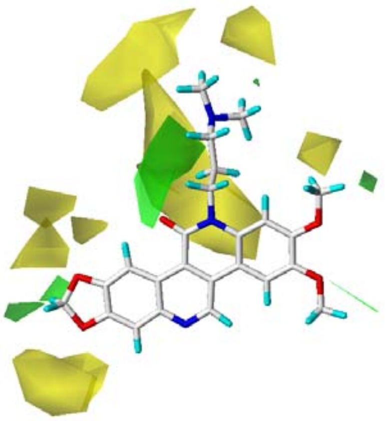

BMC_04_5585_15B

pIC_{50} pred (exp): 1,709 (3,301)

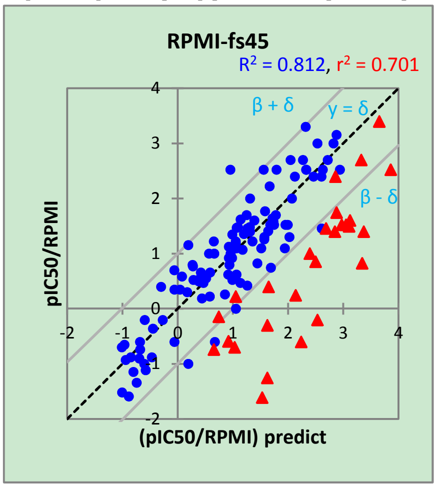

Model 19. 3D-QSAR on CPTk5 cytotoxicity

CPTk5	q^2 for each column filter values				
**Descriptor field**	1	2	3	4	5
**S**	0,308	0,308	0,304	0,301	0,302
**E**	0,222	0,221	0,218	0,207	0,204
**H**	0,283	0,287	0,288	0,269	0,262
**D**	0,000	0,000	0,000	0,000	0,006
**A**	0,068	0,071	0,077	0,067	0,064
**s,e**	0,226	0,230	0,229	0,219	0,219
**s,h**	0,326	0,333	0,336	0,338	0,335
**s,d**	-	-	-	-	-
**s,a**	-	-	-	-	-
**e,h**	0,219	0,219	0,217	0,195	0,193
**e,d**	-	-	-	-	-
**e,a**	-	-	-	-	-
**h,d**	-	-	-	-	-
**h,a**	-	-	-	-	-
**d,a**	-	-	-	-	-
**s,e,h**	0,250	0,254	0,253	0,241	0,238
**s,e,d**	-	-	-	-	-
**s,e,a**	-	-	-	-	-
**s,h,d**	-	-	-	-	-
**s,h,a**	-	-	-	-	-
**s,d,a**	-	-	-	-	-
**e,h,d**	-	-	-	-	-
**e,h,a**	-	-	-	-	-
**e,d,a**	-	-	-	-	-
**h,d,a**	-	-	-	-	-
**s,e,h,d**	-	-	-	-	-
**s,e,h,a**	-	-	-	-	-
**s,e,d,a**	-	-	-	-	-
**s,h,d,a**	-	-	-	-	-
**e,h,d,a**	-	-	-	-	-
**s,e,h,d,a**	-	-	-	-	-
**CoMFA**	0,216	0,221	0,226	0,242	0,204
**CoMFA(s)**	0,305	0,306	0,304	0,294	0,232

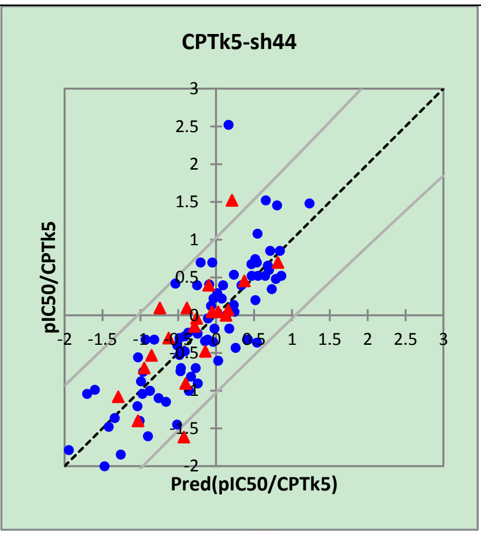

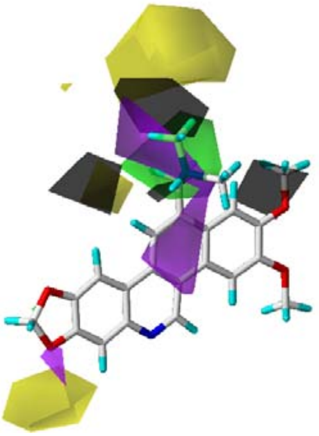

BMC_09_2877_8

pIC_{50} pred (exp): 1,230 (1,481)

Model 20. 3D-QSAR on P388 cytotoxicity

P388	q² for each column filter values				
Descriptor field	1	2	3	4	5
S	0,309	0,304	0,295	0,280	0,264
E	0,098	0,098	0,063	0,050	0,002
H	0,151	0,139	0,140	0,138	0,138
D	0,000	0,000	0,000	0,000	0,000
a	0,000	0,000	0,000	0,000	0,000
s,e	0,300	0,295	0,280	0,268	0,250
s,h	0,265	0,266	0,270	0,275	0,277
s,d	-	-	-	-	-
s,a	-	-	-	-	-
e,h	0,168	0,167	0,152	0,147	0,107
e,d	-	-	-	-	-
e,a	-	-	-	-	-
h,d	-	-	-	-	-
h,a	-	-	-	-	-
d,a	-	-	-	-	-
s,e,h	0,229	0,235	0,222	0,226	0,204
s,e,d	-	-	-	-	-
s,e,a	-	-	-	-	-
s,h,d	-	-	-	-	-
s,h,a	-	-	-	-	-
s,d,a	-	-	-	-	-
e,h,d	-	-	-	-	-
e,h,a	-	-	-	-	-
e,d,a	-	-	-	-	-
h,d,a	-	-	-	-	-
s,e,h,d	-	-	-	-	-
s,e,h,a	-	-	-	-	-
s,e,d,a	-	-	-	-	-
s,h,d,a	-	-	-	-	-
e,h,d,a	-	-	-	-	-
s,e,h,d,a	-	-	-	-	-
CoMFA	0,188	0,168	0,180	0,176	0,136
CoMFA(s)	0,293	0,293	0,293	0,289	0,283

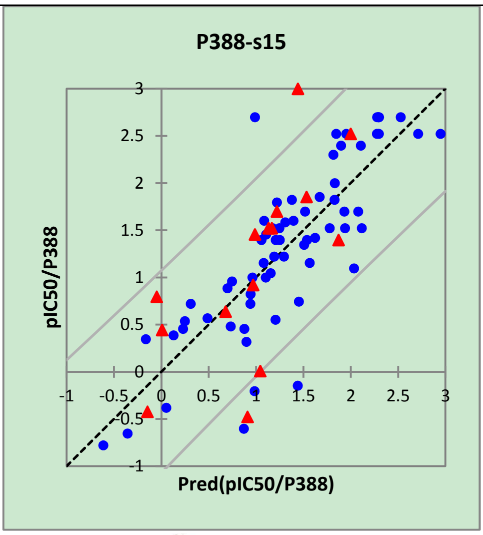

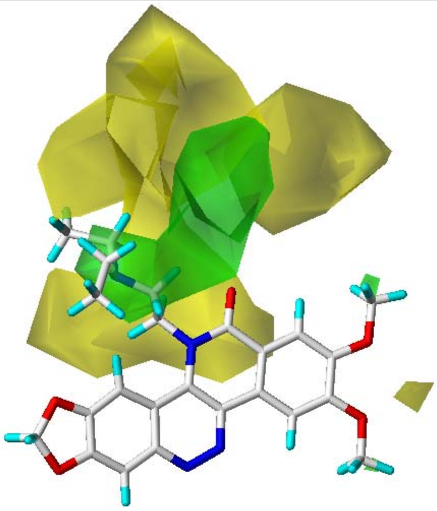

BMC_04_795_1B

pIC_{50} pred (exp): 2,699 (2,526)

Model 21. 3D-QSAR on CPT45 cytotoxicity

CPT45	q² for each column filter values				
Descriptor field	1	2	3	4	5
s	0,196	0,197	0,200	0,201	0,201
e	0,007	0,008	0,010	0,009	0,005
h	0,000	0,000	0,000	0,000	0,000
d	0,000	0,000	0,000	0,000	0,000
a	0,000	0,000	0,000	0,000	0,000
s,e	0,018	0,019	0,020	0,019	0,018
s,h	-	-	-	-	-
s,d	-	-	-	-	-
s,a	-	-	-	-	-
e,h	-	-	-	-	-
e,d	-	-	-	-	-
e,a	-	-	-	-	-
h,d	-	-	-	-	-
h,a	-	-	-	-	-
d,a	-	-	-	-	-
s,e,h	-	-	-	-	-
s,e,d	-	-	-	-	-
s,e,a	-	-	-	-	-
s,h,d	-	-	-	-	-
s,h,a	-	-	-	-	-
s,d,a	-	-	-	-	-
e,h,d	-	-	-	-	-
e,h,a	-	-	-	-	-
e,d,a	-	-	-	-	-
h,d,a	-	-	-	-	-
s,e,h,d	-	-	-	-	-
s,e,h,a	-	-	-	-	-
s,e,d,a	-	-	-	-	-
s,h,d,a	-	-	-	-	-
e,h,d,a	-	-	-	-	-
s,e,h,d,a	-	-	-	-	-
CoMFA	0,000	0,000	0,000	0,000	0,000
CoMFA(s)	0,092	0,092	0,092	0,084	0,085

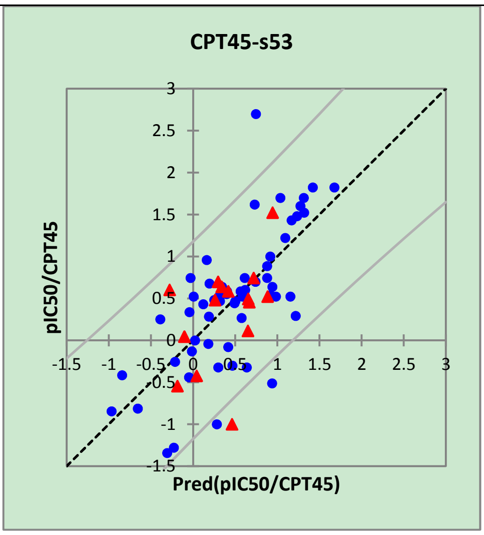

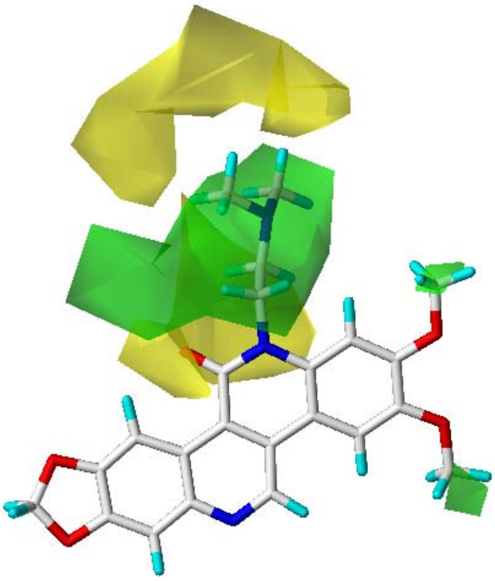

BMC_09_2877_3
pIC_{50} pred (exp): 1,418 (1,823)

Model 22-25. 3D-QSAR on KB3 cytotoxicity

KB3	q^2 for each column filter values				
Descriptor field	1	2	3	4	5
s	0,583	0,583	0,584	0,579	0,575
e	0,552	0,551	0,553	0,540	0,542
h	0,542	0,541	0,539	0,509	0,485
d	0,014	0,013	0,004	0,000	0,000
a	0,414	0,415	0,417	0,423	0,429
s,e	0,580	0,580	0,580	0,576	0,576
s,h	0,551	0,555	0,555	0,545	0,536
s,d	0,448	0,446	0,473	0,485	0,502
s,a	0,498	0,507	0,501	0,503	0,505
e,h	0,582	0,581	0,582	0,571	0,560
e,d	0,560	0,562	0,568	0,545	0,534
e,a	0,561	0,558	0,552	0,550	0,547
h,d	0,437	0,438	0,438	0,411	0,406
h,a	0,466	0,465	0,465	0,463	0,467
d,a	0,409	0,412	0,417	0,423	0,427
s,e,h	0,585	0,581	0,584	0,579	0,570
s,e,d	0,578	0,578	0,586	0,584	0,575
s,e,a	0,563	0,564	0,562	0,561	0,558
s,h,d	0,514	0,527	0,533	0,528	0,516
s,h,a	0,511	0,515	0,511	0,510	0,511
s,d,a	0,499	0,507	0,514	0,530	0,509
e,h,d	0,563	0,565	0,573	0,560	0,552
e,h,a	0,560	0,557	0,551	0,546	0,540
e,d,a	0,526	0,528	0,533	0,531	0,520
h,d,a	0,444	0,446	0,445	0,443	0,449
s,e,h,d	0,572	0,574	0,579	0,571	0,562
s,e,h,a	0,569	0,569	0,565	0,561	0,555
s,e,d,a	0,548	0,551	0,555	0,558	0,544
s,h,d,a	0,493	0,507	0,515	0,518	0,501
e,h,d,a	0,528	0,529	0,530	0,523	0,513
s,e,h,d,a	0,545	0,547	0,543	0,540	0,537
CoMFA	0,549	0,549	0,546	0,547	0,551
CoMFA(s)	0,573	0,574	0,573	0,571	0,565

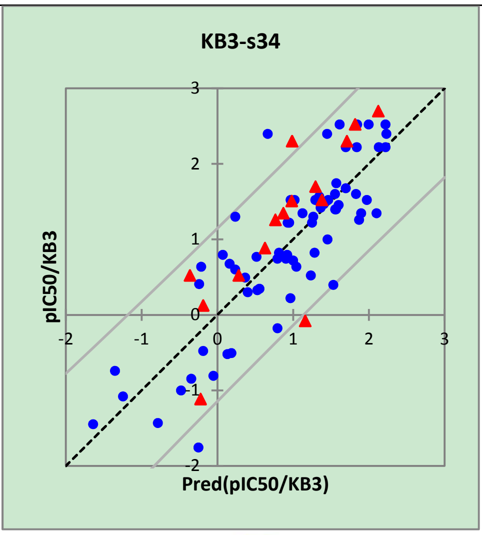

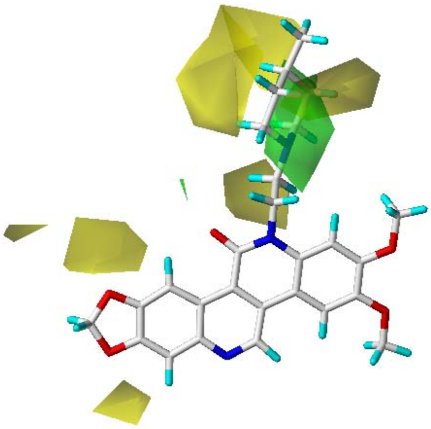

BMC_06_3131_10F
pIC_{50} pred (exp): 2,229 (2,398)

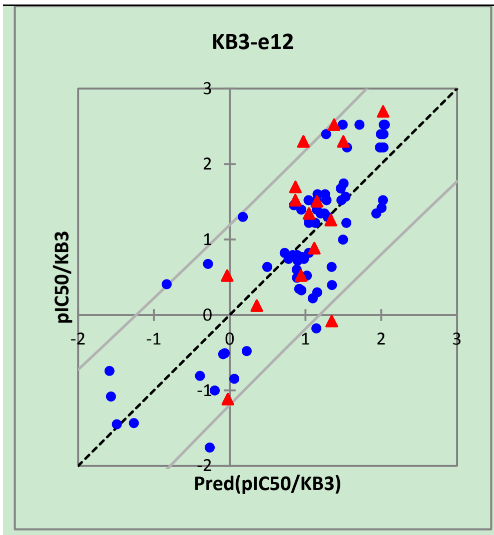

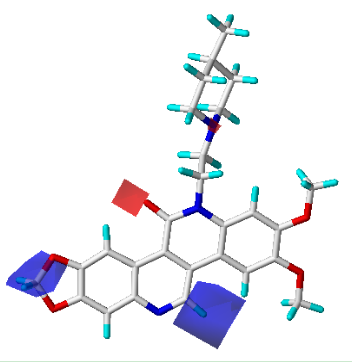

BMC_06_3131_10F
pIC50 pred (exp): 2,229 (2,398)

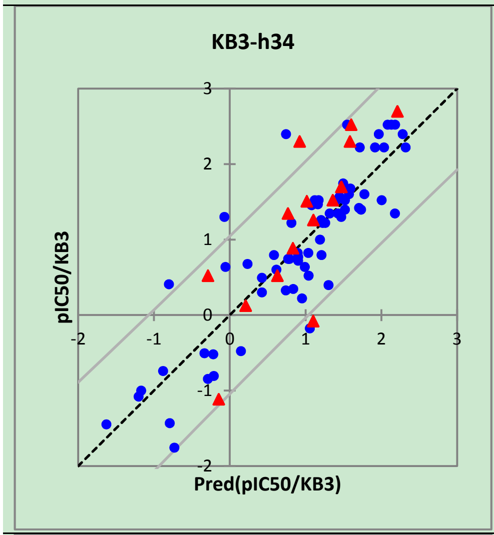

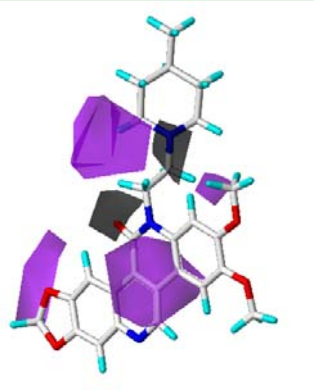

BMC_06_3131_10F
pIC50 pred (exp): 2,280 (2,398)

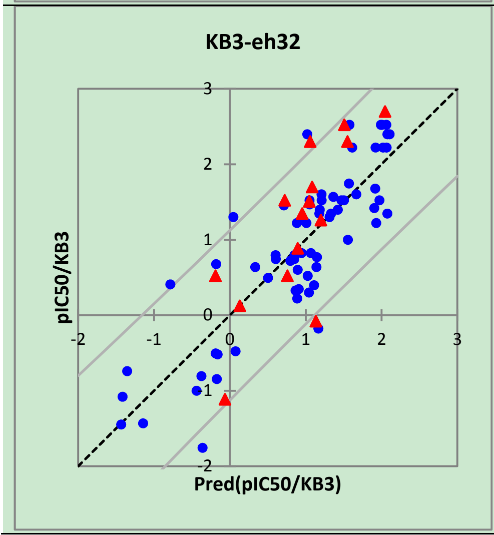

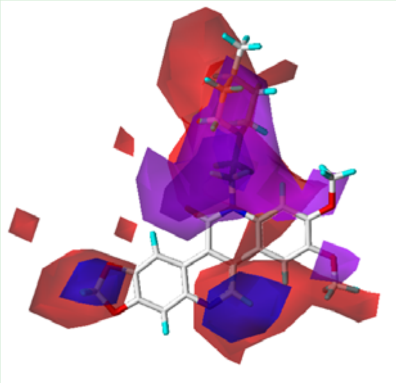

BMC_06_3131_10F
pIC50 pred (exp): 2,280 (2,398)

.

Model 26. 3D-QSAR on KBV cytotoxicity

KBV	q^2 for each column filter values				
Descriptor field	1	2	3	4	5
S	0,370	0,369	0,372	0,367	0,357
E	0,347	0,347	0,341	0,340	0,314
H	0,225	0,224	0,224	0,219	0,219
D	0,000	0,000	0,000	0,000	0,006
a	0,199	0,198	0,200	0,209	0,225
s,e	0,342	0,342	0,341	0,334	0,302
s,h	0,270	0,274	0,275	0,267	0,255
s,d	-	-	-	-	-
s,a	0,351	0,362	0,364	0,360	0,375
e,h	0,324	0,322	0,319	0,302	0,284
e,d	-	-	-	-	-
e,a	0,315	0,316	0,306	0,303	0,277
h,d	-	-	-	-	-
h,a	0,249	0,249	0,246	0,242	0,242
d,a	-	-	-	-	-
s,e,h	0,327	0,327	0,325	0,313	0,286
s,e,d	-	-	-	-	-
s,e,a	0,306	0,299	0,296	0,285	0,266
s,h,d	-	-	-	-	-
s,h,a	0,259	0,263	0,253	0,250	0,247
s,d,a	-	-	-	-	-
e,h,d	-	-	-	-	-
e,h,a	0,294	0,292	0,280	0,274	0,268
e,d,a	-	-	-	-	-
h,d,a	-	-	-	-	-
s,e,h,d	-	-	-	-	-
s,e,h,a	0,310	0,308	0,296	0,278	0,270
s,e,d,a	-	-	-	-	-
s,h,d,a	-	-	-	-	-
e,h,d,a	-	-	-	-	-
s,e,h,d,a	-	-	-	-	-
CoMFA	0,356	0,350	0,323	0,321	0,307
CoMFA(s)	0,370	0,369	0,372	0,367	0,357

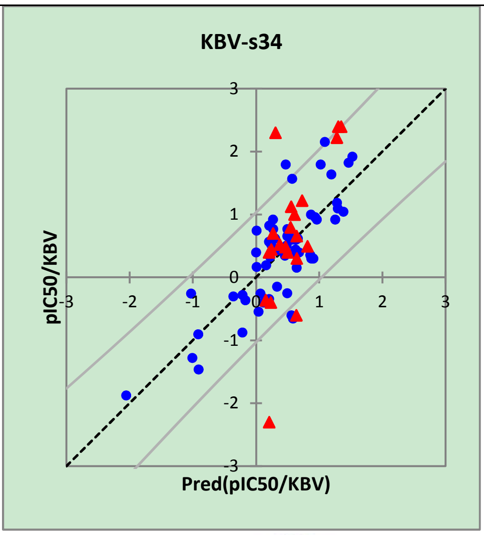

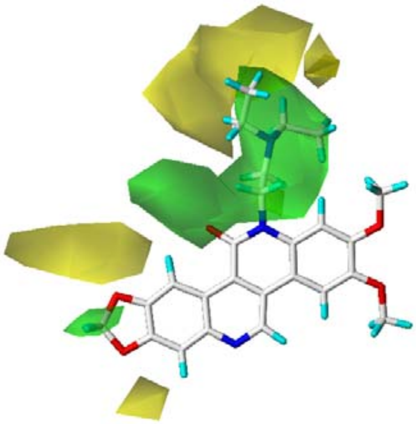

BMC_06_3131_10C
pIC_{50} pred (exp):
1,523 (1,920)

Model 27. 3D-QSAR on KBH cytotoxicity

KBH	q^2 for each column filter values				
Descriptor field	1	2	3	4	5
s	0,277	0,273	0,267	0,257	0,249
e	0,247	0,243	0,312	0,281	0,239
h	0,219	0,219	0,219	0,218	0,220
d	0,000	0,000	0,000	0,000	0,000
a	0,217	0,217	0,219	0,221	0,217
s,e	0,243	0,238	0,240	0,239	0,231
s,h	0,208	0,203	0,200	0,199	0,197
s,d	-	-	-	-	-
s,a	0,215	0,212	0,211	0,226	0,203
e,h	0,264	0,265	0,268	0,268	0,266
e,d	-	-	-	-	-
e,a	0,221	0,216	0,224	0,230	0,219
h,d	-	-	-	-	-
h,a	0,219	0,217	0,218	0,220	0,219
d,a	-	-	-	-	-
s,e,h	0,255	0,254	0,256	0,255	0,251
s,e,d	-	-	-	-	-
s,e,a	0,217	0,212	0,218	0,223	0,213
s,h,d	-	-	-	-	-
s,h,a	0,221	0,219	0,220	0,221	0,219
s,d,a	-	-	-	-	-
e,h,d	-	-	-	-	-
e,h,a	0,232	0,231	0,234	0,235	0,233
e,d,a	-	-	-	-	-
h,d,a	-	-	-	-	-
s,e,h,d	-	-	-	-	-
s,e,h,a	0,234	0,233	0,235	0,237	0,234
s,e,d,a	-	-	-	-	-
s,h,d,a	-	-	-	-	-
e,h,d,a	-	-	-	-	-
s,e,h,d,a	-	-	-	-	-
CoMFA	0,138	0,129	0,141	0,154	0,169
CoMFA(s)	0,328	0,329	0,328	0,330	0,332

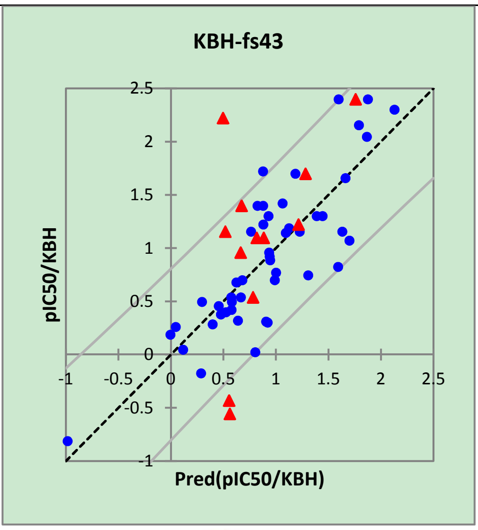

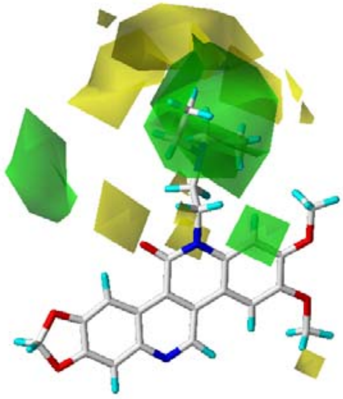

BMC_06_3131_10C
pIC_{50} dự đoán (thực nghiệm):
2,129 (2.301)

Model 28-29. 3D-QSAR on Topoisomerase -1 inhibitory activity

TOP1	q^2 for each column filter values				
Descriptor field	1	2	3	4	5
s	0,425	0,423	0,423	0,379	0,379
e	0,287	0,289	0,289	0,280	0,267
h	0,340	0,340	0,341	0,346	0,341
d	0,126	0,108	0,077	0,038	0,019
a	0,113	0,112	0,096	0,078	0,082
s,e	0,310	0,315	0,320	0,315	0,306
s,h	0,364	0,365	0,368	0,367	0,351
s,d	0,403	0,390	0,397	0,377	0,331
s,a	0,293	0,279	0,281	0,277	0,268
e,h	0,329	0,331	0,324	0,307	0,300
e,d	0,367	0,361	0,343	0,287	0,255
e,a	0,330	0,332	0,323	0,306	0,293
h,d	0,360	0,334	0,323	0,301	0,280
h,a	0,315	0,284	0,261	0,256	0,237
d,a	0,137	0,137	0,136	0,123	0,130
s,e,h	0,336	0,340	0,344	0,335	0,329
s,e,d	0,405	0,402	0,393	0,366	0,350
s,e,a	0,333	0,336	0,325	0,322	0,308
s,h,d	0,403	0,390	0,386	0,377	0,367
s,h,a	0,326	0,318	0,313	0,312	0,302
s,d,a	0,293	0,262	0,267	0,245	0,245
e,h,d	0,369	0,359	0,345	0,310	0,300
e,h,a	0,335	0,334	0,326	0,313	0,306
e,d,a	0,327	0,306	0,288	0,257	0,256
h,d,a	0,193	0,180	0,175	0,164	0,166
s,e,h,d	0,393	0,387	0,380	0,359	0,347
s,e,h,a	0,348	0,349	0,345	0,338	0,327
s,e,d,a	0,370	0,353	0,343	0,322	0,315
s,h,d,a	0,296	0,273	0,279	0,267	0,272
e,h,d,a	0,319	0,305	0,294	0,267	0,275
s,e,h,d,a	0,351	0,341	0,339	0,319	0,322
CoMFA	0,292	0,298	0,303	0,324	0,307
CoMFA(s)	0,366	0,366	0,360	0,348	0,343

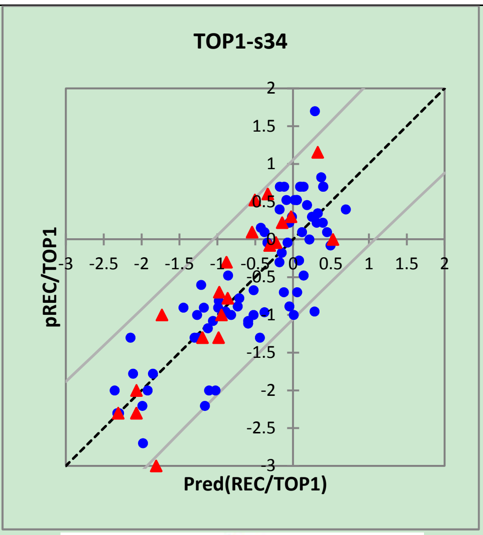

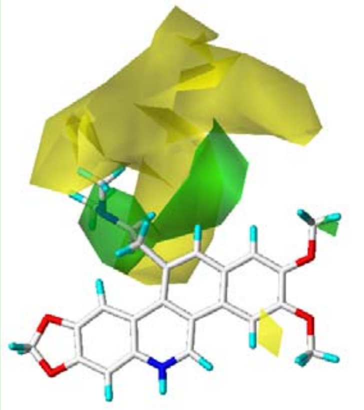

BMC_08_8598_17
pIC50 pred (exp)
0,694 (0,398)

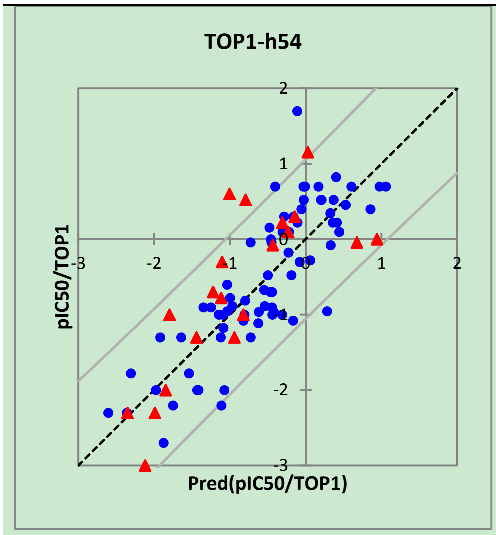

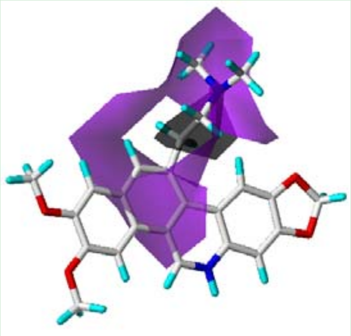

BMC_08_8598_17
pIC50 pred (exp)
0,694 (0,816)

Table S10. Several new designed BCPs compound with predictive activity from QSAR models

	Predicted pIC50 RPMI cytotoxicity			Predicted pIC50 KB3 cytotoxicity			Predicted pREC (TopI)		
Chemical structure	2D-QSAR	H-QSAR	3D-QSAR	2D-QSAR	H-QSAR	3D-QSAR	2D-QSAR	H-QSAR	3D-QSAR
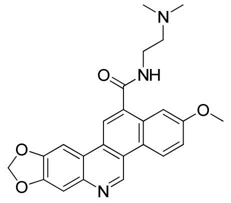	1.988	1.524	1.674	0.762	0.890	1.237	0.233	-0.689	-0.557
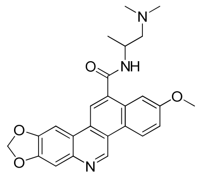	1.749	1.533	1.870	0.882	0.963	0.657	0.230	-0.623	-1.208
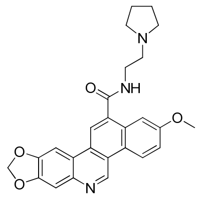	1.208	1.654	1.669	1.010	0.863	1.298	0.298	-0.533	-0.539
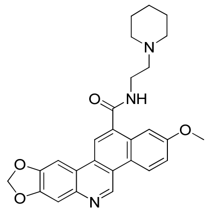	1.822	1.754	1.747	1.370	0.865	1.364	-0.046	-0.505	-0.169
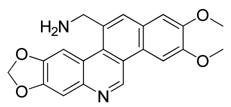	0.613	0.679	0.705	0.941	0.881	0.856	0.095	0.080	-0.052
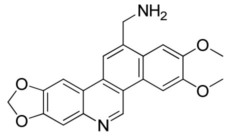	0.542	0.391	1.060	0.940	0.663	0.925	-0.151	-0.300	-0.196
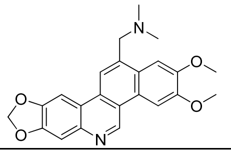	0.814	0.492	1.254	0.880	0.761	1.403	-0.432	-0.278	0.083

Part S. Calculation of r_m^2 metrics

Determination coefficient r_m^2 for the least squares regression line (without intercept) correlating observed (y-axis) and predicted (x-axis) values proposed by Roy *et al.* [1-3] was also applied to validate our QSAR models. This metric is calculated based on the correlations between the observed and predicted values with (r^2) and without (r_0^2) intercept for the least squares regression lines as shown in the following equation:

$$r_m^2 = r^2 \times (1 - \sqrt{r^2 + r_0^2} \quad (1)$$

Average of r_m^2 and $r_m^{/2}$ are performed by $\overline{r_m^2} = \frac{r_m^2 + r_m^{/2}}{2}$ with r_m^2 is validation metric calculated according to Eq. (1) using observed (y-axis) and predicted (x-axis) values; and $r_m^{/2}$ is validation metric calculated according to Eq. (1) using observed (x-axis) and predicted (y-axis) values. Absolute difference between r_m^2 and $r_m^{/2}$ was also calculated by $r_m^2 = r_m^2 - r_m^{/2}$ [1-3].

References

1. Roy, K.; Mitra, I.; Kar, S.; Ojha, P.K.; Das, R.N.; Kabir, H. Comparative Studies on Some Metrics for External Validation of QSPR Models. *J. Chem. Inf. Model.* **2012**, *52,* 396−408
2. Ojha, P.K.; Mitra, I.; Das, R.N.; Roy, K. Further exploring r_m^2 metrics for validation of QSPR models. *Chemometr. Intell. Lab.* **2011**, *107,* 194–205.
3. Pratim Roy, P.; Paul, S.; Mitra, I.; Roy, K. On Two Novel Parameters for Validation of Predictive QSAR Models. *Molecules* **2009**, *14*, 1660–1701.
